# Nitrogen Fixation and Molecular Oxygen: Comparative Genomic Reconstruction of Transcription Regulation in *Alphaproteobacteria*

**DOI:** 10.3389/fmicb.2016.01343

**Published:** 2016-08-26

**Authors:** Olga V. Tsoy, Dmitry A. Ravcheev, Jelena Čuklina, Mikhail S. Gelfand

**Affiliations:** ^1^Research and Training Center on Bioinformatics, A.A. Kharkevich Institute for Information Transmission Problems, Russian Academy of SciencesMoscow, Russia; ^2^Luxembourg Centre for Systems Biomedicine, University of LuxembourgEsch-sur-Alzette, Luxembourg; ^3^Moscow Institute of Physics and TechnologyDolgoprudny, Russia; ^4^Faculty of Bioengineering and Bioinformatics, Moscow State UniversityMoscow, Russia; ^5^Skolkovo Institute of Science and TechnologySkolkovo, Russia; ^6^Faculty of Computer Science, Higher School of EconomicsMoscow, Russia

**Keywords:** bacteria, transcription factors, nitrogen fixation, comparative genomics, regulons

## Abstract

Biological nitrogen fixation plays a crucial role in the nitrogen cycle. An ability to fix atmospheric nitrogen, reducing it to ammonium, was described for multiple species of *Bacteria* and *Archaea*. The transcriptional regulatory network for nitrogen fixation was extensively studied in several representatives of the class *Alphaproteobacteria*. This regulatory network includes the activator of nitrogen fixation NifA, working in tandem with the alternative sigma-factor RpoN as well as oxygen-responsive regulatory systems, one-component regulators FnrN/FixK and two-component system FixLJ. Here we used a comparative genomics approach for *in silico* study of the transcriptional regulatory network in 50 genomes of *Alphaproteobacteria*. We extended the known regulons and proposed the scenario for the evolution of the nitrogen fixation transcriptional network. The reconstructed network substantially expands the existing knowledge of transcriptional regulation in nitrogen-fixing microorganisms and can be used for genetic experiments, metabolic reconstruction, and evolutionary analysis.

## Introduction

Nitrogen is indispensable for all living species. The Earth atmosphere mainly consists of nitrogen, but in the form of dinitrogen (N_2_) which is not available for living organisms. Only some *Bacteria* and *Archaea* can convert N_2_ into ammonium (NH_3_) to further synthesize organic compounds. This process is known as biological nitrogen fixation. Biological nitrogen fixation plays a crucial role in the nitrogen cycle as it returns the element from the geosphere and atmosphere to living species (reviewed in Dixon and Kahn, 2004).

Species capable of nitrogen fixation belong to *Bacteria* and *Archaea*, as no examples of nitrogen fixation in *Eukarya* are known. Within *Archaea*, nitrogen fixation has been observed in some methanogens (*Methanobacteriales, Methanococcales*, and *Methanosarcinales*). In *Bacteria*, nitrogen fixation is much more widely distributed and has been characterized in phyla *Actinobacteria, Bacteroidetes, Cyanobacteria, Chlorobi, Chloroflexi, Firmicutes*, and *Proteobacteria* (Raymond et al., 2004; Dos Santos et al., 2012; Boyd and Peters, 2013).

Nitrogen fixation is often the limiting factor for crop and natural ecosystem productivity making this process important for agriculture (Dixon and Kahn, 2004). It is carried out that a large number of nitrogen fixing bacteria in soils and in symbioses with plants are *Alphaproteobacteria*.

The main enzyme catalyzing reduction of N_2_ to ammonium is the nitrogenase. All known nitrogenases require a FeS-cluster and other metal-dependent cofactors for electron transduction. The best studied and the most common is the molybdenum-dependent nitrogenase which is encoded by the *nifHDK* genes (Boyd and Peters, 2013). Alternative nitrogenases are the vanadium- and iron-dependent nitrogenases encoded by the *vnfHDGK* and *anfHDGK* genes, respectively (Rehder, 2000; Seefeldt et al., 2009; Hartmann and Barnum, 2010). Since nitrogenases are irreversibly inhibited by dioxygen (O_2_) (Gallon, 1981; Boyd and Peters, 2013), bacteria developed various strategies to avoid the latter, such as elimination of O_2_ through enzyme-catalyzed reactions, symbiotic nodules, compartmentalization etc (Oelze, 2000; Boyd et al., 2015).

In spite of the importance of nitrogen fixation, the regulation of this process has been well described in only a few bacterial species. Diazotrophs have developed multiple strategies in regulation of nitrogen fixation. Some regulate nitrogen fixation post-translationally (Huergo et al., 2012). For example, in *Rhodobacter capsulatus* nitrogenase can be by reversibly inactivated by the DraT protein (Masepohl et al., 2002). Proteins NifL and NifI_1_I_2_, involved in post-translational regulation in *Klebsiella pneumoniae* (Dixon and Kahn, 2004) and *Heliobacterium chlorum* (Enkh-Amgalan et al., 2006) respectively, are absent in *Alphaproteobacteria* (Boyd et al., 2015). PII proteins, critical for *Herbaspirillum seropedicae*, have been shown to be non-essential for nitrogen fixation in some *Alphaproteobacteria* (Arcondeguy et al., 1997; Yurgel et al., 2010).

From early studies on nitrogen fixation in rhizobia it is known, that nitrogen fixation cannot occur in *nifA, fixJ*, and *fixK* mutants. All these genes encode transcription factors (Hennecke, 1990). In *Proteobacteria*, the nitrogenase genes are invariably activated by NifA, while FixJ and FixK were shown to control nitrogen fixation in response to microaerobic conditions in certain *Rhizobiales* (Fischer, 1994). The regulation by NifA, FixJ, and FixK has been well described in only a few model species. Whether functions regulated by these factors span beyond regulation of nitrogen fixation, and what are other possible members of this regulatory network, what are the relationships between these regulators, and to what extent these networks are conserved in different species is not clear.

In most nitrogen-fixing bacteria NifA is the master regulator of nitrogen fixation. It works in association with the RNA-polymerase sigma factor RpoN (sigma54; Sullivan et al., 2002; Sciotti et al., 2003). RpoN recognizes a -24/-12 promoter sequence with the consensus TGGCACG-N_4_-TTGCW and binds the NifA protein which in turn recognizes its own binding site with the consensus sequence TGT-N_10_-ACA (Barrios et al., 1999). As mentioned above, nitrogen fixation is very sensitive to O_2_ concentration, and NifA has an ability to sense it through conserved cysteine residues. While NifA effects nitrogen fixation itself, FixLJ and FnrN/FixK are responsible for the cell adaptation to microaerobic conditions (Sciotti et al., 2003).

FixJ is a DNA-binding response regulator working in tandem with sensor kinase FixL, constituting a two-component regulatory system FixLJ (Reyrat et al., 1993; Torres et al., 2011). FixL directly senses O_2,_ which binds the haem group in the sensor domain and inactivates its kinase activity (Rey and Harwood, 2010; Sousa et al., 2015). FixJ is one of the major regulators of nitrogen fixation, but the only known conserved member of the FixJ regulon is *fixK* (Nellen-Anthamatten et al., 1998). Several attempts have been made to determine its binding motif, leading to varying results. Sequence analysis of the *fixK* promoter region in *Sinorhizobium. meliloti, Azorhizobium caulinodans* and *Bradyrhizobium japonicum* yielded a common CSNAATWT motif at position -33 and an additional TAAG element around position -64 (Sciotti et al., 2003). This observation was confirmed by DNase protection which showed that *S. meliloti* FixJ binds to the *fixK* promoter in two different regions (-69, -44) and (-57 and -31) relative to the transcription start site (Galinier et al., 1994). The application of the SELEX technique predicted two classes of FixJ-binding sites with the consensus motifs GTAGTTTCCC and GTAMGTAG (Ferrieres and Kahn, 2002). The most recent studies analyzed FixJ-binding sites in *S. meliloti* using a gel shift assay and the NMR structure of the truncated C-terminal FixJ DNA-binding domain. They determined the TAAGTAATTTCCCTTA sequence in the upstream region of the *fixK* gene as the FixJ-binding site (Kurashima-Ito et al., 2005). Therefore, the lack of a common consensus complicates identification of new FixJ targets.

Some *Rhizobiales*, in particular, *R. etli* and *R. leguminosarum*, lack FixJ, and thus use an alternative regulatory cascade comprised of a hybrid histidine kinase hFixL and a response regulator FxkR, analogous to FixJ and FixKf. A FxkR-binding site with the consensus GTTACA-N_4_-GTTACA was identified upstream of the *fixKf* gene by MEME and confirmed by the gel shift assay (Zamorano-Sanchez et al., 2012).

Other regulators of nitrogen fixation, FixK and FnrN, belong to the Crp-Fnr superfamily of transcription factors (Matsui et al., 2013; Rodgers et al., 2013). The main difference between these proteins is the ability of FnrN to directly sense oxygen with the iron–sulfur cluster. The cluster is formed by the conserved N-terminal cysteine-rich domain and an additional cysteine in the middle of the polypeptide chain (Korner et al., 2003). FixK and FnrN have been studied extensively in *Alphaproteobacteria* and shown to control expression of oxidases, hydrogen uptake, nitrogen metabolism, haem biosynthesis, and nitrogen-fixation transcription factors (Supplementary Table S1 in the Supplementary Materials). The existence of two closely related regulators, FnrN and FixK, is the main obstacle for the genomic analysis of their regulatory interactions, as both transcription factors recognize very similar binding sites with the consensus motif TTGANCNNGATCAANG (Cebolla and Palomares, 1994; Mesa et al., 2005, 2008; Bonnet et al., 2013).

Hence, known regulatory networks for nitrogen fixation in *Alphaproteobacteria* are rather complex, may vary between species, and seem highly redundant due to the presence of multiple transcription factors with very similar functions. The experimental data about transcriptional regulation of nitrogen fixation are difficult to obtain and thus are generated at a slow rate. On the other hand, the growing number of complete bacterial genomes and the development of comparative genomics techniques allow us to analyze transcriptional regulatory systems, addressing relevant biological questions. Here we report the results of manual comparative analysis of the regulatory network for nitrogen fixation in 50 complete genomes belonging to five orders of *Alphaproteobacteria*. We predict new members of the nitrogen fixation regulatory network and reconstruct different types of regulatory cascades found in *Alphaproteobacteria*. Based on these predictions, we propose a model of the gradual growth of complexity for this network and reconstruct possible evolutionary events that have formed the network. We also have constructed taxon-specific profiles for each of the analyzed regulators. The obtained dataset including the information about the analyzed transcription regulators, their binding sites and motifs, and regulated genes is deposited in the RegPrecise database (Novichkov et al., 2013).

## Materials and Methods

### Comparative Genomics Approach for the Reconstruction of Regulons

For the regulon reconstruction, we used a comparative genomics approach (Rodionov, 2007) implemented in the RegPredict Web server (Novichkov et al., 2010). The workflow includes inference of transcription regulator binding sites, construction of positional weight matrices (profiles) for the binding-site motifs, and further search for additional regulon members based on predicted binding sites in gene promoter regions. To take into account possible lineage-specific changes in binding site motifs, we constructed individual profiles for genomes from each considered order of *Alphaproteobacteria*.

To construct the profiles, the following steps were implemented. An initial profile was constructed based on known binding sites of a given regulator or on sequences of binding sites predicted by phylogenetic footprinting, an approach based on the analysis of conserved islands in multiple alignments of DNA fragments (Shelton et al., 1997; McCue et al., 2001). This profile was used for site prediction in genomes from a given order. Two types of predicted sites were used to update the taxon-specific profile: (1) sites predicted upstream of orthologs of genes known to be regulated in at least one genome and (2) sites upstream of operons with conserved predicted regulation, i.e., sites that were found upstream of orthologous genes in at least than half of genomes from the analyzed order (the consistency check approach; Ravcheev et al., 2007; Rodionov, 2007). Operons were defined as groups of genes satisfying the following criteria: same direction of transcription, intergenic distance not exceeding 200 bp, absence of internal binding sites, and conservation of the locus structure in a number of related genomes. The obtained order-specific profile was further used for the analysis of regulons in this order.

The initial profiles for the NifA and FnrN/FixK-like proteins were constructed based on sequences of known binding sites extracted from literature data. For the FixJ and FxkR proteins, the initial profiles were constructed using phylogenetic footprinting. The constructed profiles were further used to search for additional regulon members using the Run Profile tool in RegPredict. The lowest score observed in the training set of known and/or initially predicted binding sites was used as the threshold for the genome-wide site search. To eliminate false positives, the consistency check approach or functional relevance of candidate target operons were used.

### Tools and Databases

Fifty studied genomes were downloaded from the MicrobesOnline database (Dehal et al., 2010). The initial search for FnrN and FixK homologs was done using BLASTP (Altschul et al., 1997) with known FnrN/FixK proteins (cutoff: *e*-value = e-30, identity = 40%). The domain structure of defined homologs was predicted with the Pfam database (Finn et al., 2014) and used as an additional criterion: a protein should the have N-terminal cyclic nucleotide-binding domain (PF00027) and C-terminal Crp-like helix-turn-helix domain (PF13545). Gene orthology was determined by the bidirectional best hit criterion implemented in the GenomeExplorer software (Mironov et al., 2000) and validated by phylogenetic trees from the MicrobesOnline database. Genes were considered as orthologs if they: (1) formed a monophyletic branch in the phylogenetic tree; and (2) demonstrated conserved chromosomal gene context. Functional gene annotations were extracted from the literature and uploaded from the SEED (Disz et al., 2010), UniProt (Magrane and Consortium, 2011), MicrobesOnline, and KEGG (Kanehisa et al., 2014) databases.

Multiple alignments for amino acid and nucleotide sequences were constructed using a web version^1^ of the MUSCLE tool (Edgar, 2004) with default parameters. Phylogenetic trees were constructed by the maximum-likelihood method (Felsenstein, 1996) and LG model for amino acid substitution (Le and Gascuel, 2008) implemented in -3.0 (Guindon et al., 2010) with default parameters, and visualized with the Dendroscope (version 3.2.10, built 19) program (Huson et al., 2007). Sequence logos for DNA motifs were drawn with WebLogo (Crooks et al., 2004).

### Data Availability

All predicted regulons including transcription regulators, their binding sites, their regulated genes and operons, and functional gene assignments are deposited in the RegPrecise database (Novichkov et al., 2013) and are freely available at http://regprecise.lbl.gov/RegPrecise (Supplementary Table S2 in the Supplementary Materials).

## Results

### Distribution of Genes Encoding the Nitrogenase and the Transcription Regulators

To describe the transcriptional regulation for nitrogen fixation systematically among *Alphaprpteobacteria*, we started with the analysis of the distribution of genes encoding the nitrogenase and the regulatory proteins in 50 genomes of *Alphaproteobacteria* (Supplementary Table S3 in the Supplementary Materials).

#### nifHDK

The nitrogenase-encoding genes *nifHDK* were found in most *Rhizobiales* and *Rhodospirillales*, and were not found in *Caulobacteriales*. The *nifHDK* genes were also detected in one *Rhodobacterales* genome, *Rhodobacter sphaeroides* 2.4.1, and one *Sphingomonadales* genome, *Zymomonas mobilis* ZM4.

#### NifA

The orthologs of the transcription regulator NifA were found in 10 *Rhizobiales*, 6 *Rhodospirillales*, 1 *Sphingomonadales*, and 1 *Rhodobacterales* genomes. No NifA orthologs were found in *Caulobacteriales*. The NifA genes are always co-localized in genome with the *nifHDK* genes, and in 10 studied genomes the *nifA* genes are co-localized with the former (Supplementary Table S3 in the Supplementary Materials).

#### FixLJ

Orthologs of the FixLJ two-component system were identified in all four analyzed *Caulobacteriales* and in 15 of 20 analyzed *Rhizobiales*. Previously, an alternative cascade called hFixL-FxkR-FixKf has been identified in several *Rhizobiales* (Zamorano-Sanchez et al., 2012). Among genomes studied here, the orthologs of these transcription factors were found in *R. etli, R. leguminosarum*, and *S. meliloti* (Supplementary Table S3 in the Supplementary Materials).

#### FnrN/FixK

Transcription factors related to FnrN and FixK that could also be important for nitrogen fixation are distributed more widely than genes for the nitrogenase itself (Supplementary Table S3 in the Supplementary Materials). Some analyzed genomes contain multiple copies of *fnrN* and/or *fixK* as usually more than one Crp-Fnr superfamily protein per genome could be found (Vollack et al., 1999; Korner et al., 2003;Matsui et al., 2013)

**FIGURE 1:**
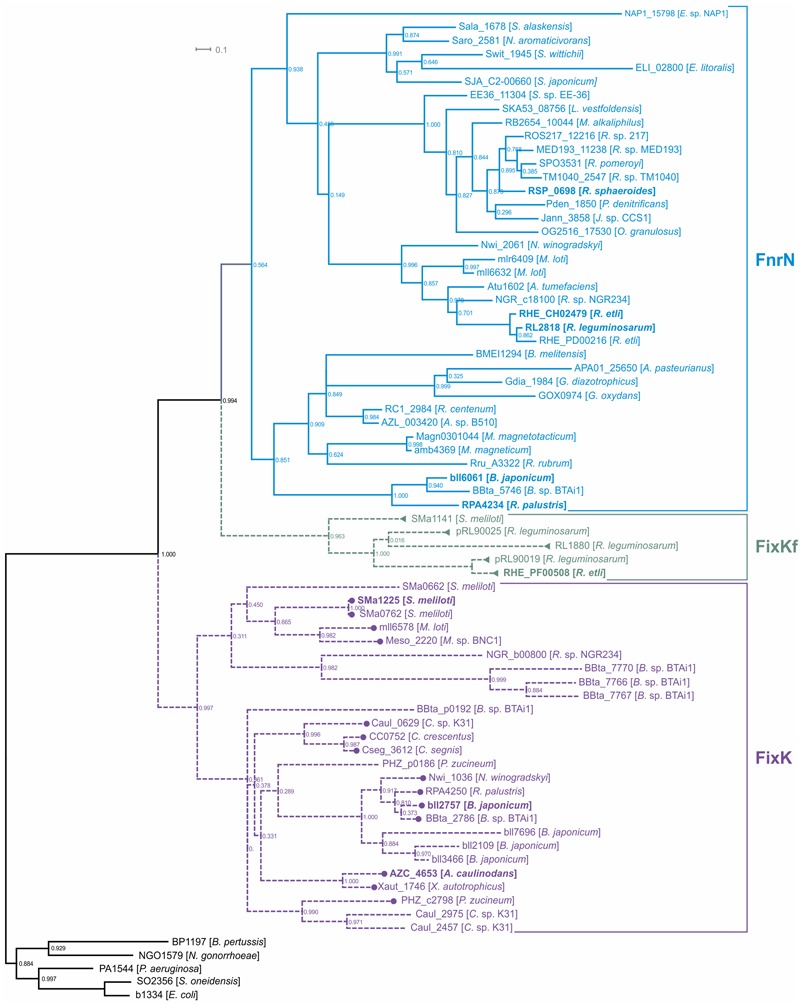
**Maximum-likelihood phylogenetic tree for FnrN/FixK-like proteins.** Locus tags for proteins are shown. Previously analyzed proteins are shown in bold. Solid branches correspond to proteins that have cysteine residues for the iron-sulfur cluster, dashed branches correspond to proteins lacking such cysteine residues. Regulation by FixJ is shown by circles, regulation by FxkR, by triangles. The following proteins were used as the outgroups: the Fnr proteins from *Bordetella pertussis* Tohama I (BP1197), *Escherichia coli K-12 MG1655* (b1334), *Neisseria gonorrhoeae* FA 1090 (NGO1579), and *Shewanella oneidensis* MR-1 (SO2356), and the Anr protein from *Pseudomonas aeruginosa* PAO1 (PA1544).

FixK and FnrN are very closely related, so we performed a detailed analysis of their orthologs, in order to be able to describe the evolution of regulatory cascades involving these factors. The FnrN/FixK orthologs were identified using the following procedure: (1) homologs of previously known FnrN/FixK were found in the analyzed genomes; (2) a phylogenetic tree for all found homologs was constructed; (3) only proteins forming monophyletic branches with known FnrN/FixK proteins were retained for further analysis (**Figure 1**; Supplementary Figure S1 in the Supplementary Materials).

As the presence of cysteine residues required for formation of oxygen-sensitive iron–sulfur clusters is a common feature of the Fnr branch of the Crp-Fnr superfamily (Korner et al., 2003; Matsui et al., 2013), to distinguish between FnrN-like and FixK-like regulators, we analyzed the detected proteins for the presence of such cysteine residues. The proteins retaining these cysteine residues formed a single monophyletic branch on the tree (**Figure 1**) and, hence, were annotated as FnrN orthologs. In addition to previously known FnrN proteins, this branch also contains the AadR protein (locus tag: *RPA4234*) from *Rhodopseudomonas palustris* (Dispensa et al., 1992; Egland and Harwood, 1999; Rey and Harwood, 2010), a transcription regulator of anaerobic degradation of aromatic acids (see below).

Proteins lacking the cysteine residues form two branches. One branch contains previously studied FixK proteins from various *Rhizobiales*, whereas the second branch includes the FixKf (locus tag: *RHE_PF00508*) protein of *R. etli* and is related to the FnrN branch (**Figure 1**). Because of that, the latter group cannot be considered as orthologs of FixK. On the other hand, the absence of cysteine residues required for formation of the iron–sulfur cluster (Supplementary Figure S1 in the Supplementary Materials) shows that these proteins are not orthologs of FnrN. As FnrN and FixKf branches are clearly separated from each other, and both types of proteins are present in *Rhizobium* spp., we propose that FnrN and FixKf are divergent paralogs.

As the FixKf branch is much closer to FnrN than to FixK, we propose that FixKf and FixK independently have lost the ability to form the iron–sulfur cluster for performing the same functions. Thus, the FixKf proteins were defined as a separate group of transcription regulators. Hence, among FnrN/FixK-like proteins at least four groups of transcription regulators can be distinguished: (1) FnrN (also known as FnrL in *R. sphaeroides*) containing an iron-sulfur cluster, (2) regulator of anaerobic degradation of aromatic acids AadR, also containing an iron-sulfur cluster, (3) FixK proteins lacking an iron-sulfur cluster, and (4) FixKf also lacking an iron-sulfur cluster, but much more closely related to FnrN than to FixK.

Overall, FnrN/FixK-like proteins were found in 43 of 50 analyzed genomes. The distribution of these proteins is taxon-specific. *Caulobacteriales* have only FixK proteins, whereas *Rhodobacterales, Rhodospirillales*, and *Sphingomonadales* have only FnrN proteins. In *Rhizobiales*, various combinations of FixK, FnrN, and FixKf were observed – only FixK in three genomes; only FnrN in two genomes; both FixK and FnrN in six genomes; FixKf and FnrN in two genomes; and FixK and FixKf in one genome (Supplementary Table S3 in the Supplementary Materials). In all studied *Alphaproteobacteria*, except *Z. mobilis*, FnrN/FixK-like proteins were found in all organisms having the nitrogenase genes.

#### FixK/FixKf and FixLJ Co-occurrence

As the FixK proteins lack the iron-sulfur cluster, they are activated in response to oxygen through the FixLJ-FixK regulatory cascade (Nellen-Anthamatten et al., 1998; Mesa et al., 2008; Reutimann et al., 2010). The *fixK* genes are present in all genomes having the *fixLJ* operon, the only exception is *Rhizobium* sp. NGR234, lacking the *fixLJ* operon. For the *fixKf* genes such correlation was not observed, as only in *S. meliloti* both *fixKf* and *fixLJ* genes were found (Supplementary Table S3 in the Supplementary Materials).

### Binding Motifs for Nitrogen-Fixation Transcription Regulators

#### NifA-Binding Motifs

The initial profile for NifA-binding sites was constructed using previously known sites in *R. etli* (Salazar et al., 2010) and *B. japonicum* (Caldelari Baumberger et al., 2003; Hauser et al., 2007). Specific profiles were constructed for all four orders having *nifA* genes. Due to the degeneracy of the NifA-binding motif (Bueno et al., 2010; Salazar et al., 2010), the obtained profiles yielded a large number of false-positives. On the other hand, since NifA acts as a transcription activator only with the RpoN sigma-factor, we used prediction of RpoN promoters to eliminate false-positive NifA-binding sites. Thus, we constructed a profile using experimentally known RpoN promoters (Caldelari Baumberger et al., 2003; Hauser et al., 2007; Salazar et al., 2010). A predicted NifA-binding site was considered as a true positive only if it had a downstream predicted RpoN-binding site (Supplementary Table S4 in the Supplementary Materials).

#### FnrN/FixK-Binding Motifs

**FIGURE 2:**
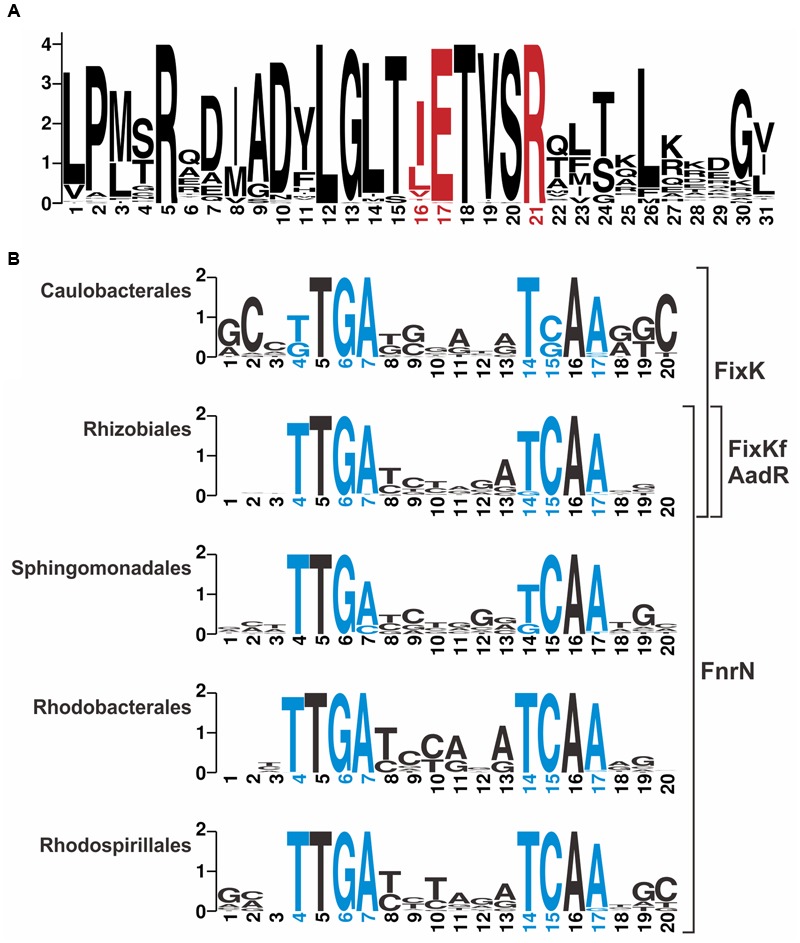
**Sequence Logos for **(A)** HTH domain of FixK/FnrN-like proteins and **(B)** their binding motifs.** Positions for amino acids and nucleotides specifically interacting with each other are shown in red and blue, respectively. **(A)** The logo was constructed based on 65 proteins from 43 genomes. **(B)** Taxon-specific logos were constructed for the following numbers of sites and genomes, *Caulobacterales*, 25 sites, 4 genomes; *Rhizobiales*, 192 sites, 14 genomes, *Sphingomonadales*, 23 sites, 5 genomes; *Rhodobacterales*, 85 sites, 12 genomes; *Rhodospirillales*, 18 sites, 8 genomes.

In an attempt to distinguish binding sites for FnrN/FixK-like regulators, we analyzed in detail sequences of these proteins and their binding sites (**Figure 2**). Amino acids involved in specific interactions with nucleotides of DNA sites (Bonnet et al., 2013) are highly conserved and no group-specific changes were found (**Figure 2A**; Supplementary Figure S1 in the Supplementary Materials). Thus, we constructed taxon-specific profiles for FixK in *Caulobacteriales* and FnrN in *Rhodobacterales, Rhodospirillales*, and *Sphingomonadales* (**Figure 2B**). For *Rhizobiales*, we constructed one general profile to search for binding sites of all four regulators.

#### FixJ-Binding Motif

**FIGURE 3:**
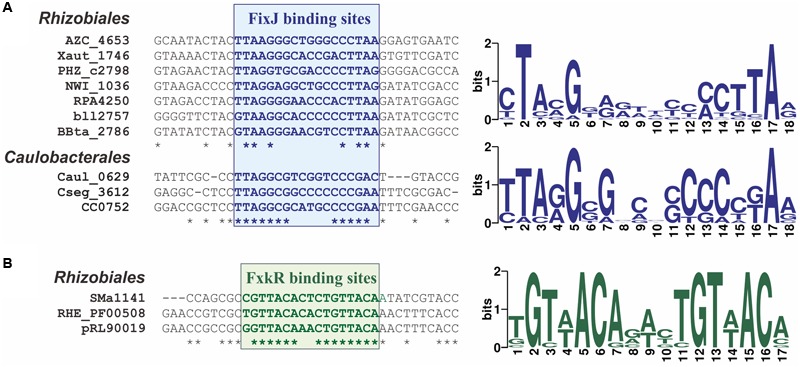
**Predicted binding sites for **(A)** FixJ and **(B)** FxkR proteins.** Fragments of alignments for **(A)** *fixK* (10 genomes) and **(B)** *fixKf* (3 genomes) upstream regions (left) and sequence logos for binding motifs (right) are shown.

Since the *fixK* gene is the main target of FixJ-dependent regulation, to determine a profile for FixJ-binding sites, we analyzed upstream regions of the *fixK* genes. Phylogenetic footprinting revealed similar 18-bp imperfect palindromes in promoter regions from both *Rhizobiales* that coincides with the previously experimentally determined 16-bp sequence (Kurashima-Ito et al., 2005) and *Caulobacteriales* (**Figure 3**; Supplementary Figure S2 in the Supplementary Materials). Order-specific profiles were constructed for FixJ-binding motifs in *Rhizobiales* and *Caulobacteriales*.

#### FxkR-Binding Motif

To build a profile for the FxkR-binding motif, we applied the phylogenetic footprinting approach to *fixKf* upstream regions. We identified a 17-bp palindromic motif that is similar to the 16-bp motif determined previously (**Figure 3**; Supplementary Figure S2 in the Supplementary Materials) (Zamorano-Sanchez et al., 2012). Site search in FxkR-containing genomes revealed putative FxkR-binding sites upstream of not only *fixKf*, but also several other relevant genes (see below).

### Structure of the Analyzed Regulons: New Members

The regulatory network for nitrogen fixation was reconstructed in 44 *Alphaproteobacteria* genomes having at least one of the analyzed regulators.

#### NifA Regulon

Genes encoding the NifA protein were found in 18 of 50 analyzed genomes. In each of these genomes NifA-binding sites were found upstream of multiple operons. In total, we found candidate NifA-binding sites upstream of 95 operons comprised of 280 genes. These genes include previously known targets such as the *nif, fix, hup* operons, *groESL, iscN, fdxN*, and *rpoN* (Supplementary Table S1 in the Supplementary Materials). In *Rhodospirillales*, we observed candidate NifA-binding sites upstream of 34 operons comprised of 165 genes. In *Rhodobacterales* and *Sphingomonadales*, we studied single nitrogen-fixing representatives, *R. sphaeroides* and *Z. mobilis*, respectively. *R. sphaeroides* has NifA-binding sites upstream of 26 genes from 7 operons, in *Z. mobilis* we predicted 24 genes from 4 operons to be under control of NifA. NifA regulation outside *Rhizobiales* has been experimentally studied only in *A. brasilense*, where it controls the expression of the *nifE* gene (Potrich et al., 2001). However, most targets predicted to be under the NifA control in *Rhodospirillales, Rhodobacterales*, and *Sphingomonadales* (such as *nif, fix, icsN*, and *fdxN* genes) have been experimentally shown to be under the NifA regulation in *Rhizobiales*.

Several new NifA regulon members were identified. The first group of genes is involved in the adaptation to microoxic conditions. It includes genes encoding cytochrome P450 (*cpx*), oxidoreductase (*fixR*), that are under NifA regulation in several *Rhizobiales*; and alkyl hydroperoxide reductases (*ahpC1, ahpC*, and *ahpD*), which have candidate NifA-binding sites in some *Rhizobiales* and *R. rubrum* ATCC 11170. The second group includes the molybdenum transport genes *modABCD*. NifA-dependent regulation of these genes is important because the molybdenum cofactor is essential for functioning of the nitrogenase protein complex. The *modABC* operon is under NifA regulation in *Bradyrhizobium* spp., *Rhizobium sp.* NGR234, and *Gluconacetobacter diazotrophicus*, whereas *modD* has NifA-binding sites in *G diazotrophicus, R. sphaeroides*, and *Z. mobilis*. The third heterogeneous group includes genes directly or indirectly involved in electron transfer. In *Rhizobiales*, ferredoxins *fdxB, fxrA, fer2*, Fe-binding protein *hesB* and Fe-responsive regulator *irr* have candidate NifA-binding sites. In *Rhodospirillales, fer2* also has a candidate NifA-binding site, and the electron transport complex *rnfABCDGEH* is also under NifA control. In *Azospirillum* sp. B510, ferredoxin-encoding *fdxB* is located in a NifA-regulated operon. In *Rhodobacterales* and *Sphingomonadales*, the *rnf* operon also has candidate NifA-binding sites. The last group is represented in *Magnetospirillum* and *Rhodospirillum*, where candidate NifA-binding sites were found upstream of the *draTG* genes that are known to switch off the nitrogenase activity by covalently binding ADP-ribose (Moure et al., 2015).

We have observed conserved NifA-binding sites upstream of several genes whose connection to nitrogen fixation is not known. NifA-binding sites were found upstream of genes encoding stress-response transcription regulator (*bolA*), xanthine dehydrogenase (*xdhC*), and outer membrane protein (*ompW*). At that, the latter was shown experimentally to be regulated by NifA in *Mesorhizobium loti* (Sullivan et al., 2013).

#### FixJ Regulon

Candidate FixJ-binding sites in *Rhizobiales* were found upstream of four genes, transcription regulators *nifA* and *fixK*, ferredoxin-like protein *fixX*, and L-proline 3-hydroxylase *SMc03253*. The regulation of all these genes except *fixX* has been previously shown experimentally (Supplementary Table S1 in the Supplementary Materials). A new target gene, *fixX*, is located in a regulated operon with *nifA* and is a part of the electron transfer *fixABCX* complex. In *Caulobacteriales*, conserved FixJ-binding sites were consistently found upstream of only one already known target, *fixK*.

#### FxkR Regulon

We also characterized the FxkR regulon in *Rhizobiales*. Its candidate binding sites were found upstream of 10 operons comprised of 21 genes including previously known target, *fixKf*, and several new targets such as *fxkR* itself and the *cco* operon.

#### FnrN/FixK Regulon

As mentioned above, FnrN/FixK-like regulators have taxon-specific distribution. In *Caulobacteriales*, only FixK has been found and here it controls the expression of 70 genes from 21 operons. The function of these targets is limited to redox processes. They include previously known targets such as cytochrome oxidase *cco* and *cyd* operons, haem biosynthesis gene *hemN*, two-component system *fixLJ*, and the *fixK* gene itself. We also observed candidate FixK-binding sites upstream of new target genes involved in redox processes, cytochrome *c* oxidase (*ctaCDBXGE*).

Only FnrN has been found in *Rhodobacterales, Rhodospirillales*, and *Sphingomonadales*. In *Rhodobacterales*, 171 genes from 71 operons are under the FnrN control, in *Rhodospirillales*, 21 genes from 17 operons, and in *Sphingomonadales*, 52 genes from 18 operons. Here the main function of the target genes is also limited to redox processes. Previously known targets include cytochrome oxidases (the *cco* operons), haem biosynthesis genes *hemA* and *hemN*, and the regulator *fnrN* genes. Newly found targets are also involved in redox processes. In *Magnetospirillum* spp., FnrN controls expression of cytochrome *c4* precursor gene *cycA* and in several *Rhodobacterales*, cytochrome c551 peroxidase *ccpR*.

*Rhizobiales* employ the most complicated FnrN/FixK-regulatory network and here the function of target genes is not limited to redox processes. Among genes involved in redox processes, FnrN/FixK-binding sites were found upstream of known targets such as the *cco, cyd* operons, haem formation genes *hemA, hemC*, and *hemN*, and some new ones, the *cta* operon, NADH-ubiquinone oxidoreductase (*nuoABCDEFGHIJKLMN*), decaheme cytochrome *c* (*mtrA*), haem lyase operon *ccmIEFH* responsible for haem incorporation into cytochrome oxidase. In *R. palustris*, FnrN/FixK-binding sites are located upstream of a gene for Fe-responsive regulator *irr*, implicating the wider role for these proteins in the regulation of redox processes.

Other FnrN/FixK targets play roles in various nitrogen metabolic processes other than nitrogen fixation as shown experimentally for *B. japonicum* (Supplementary Table S1 in the Supplementary Materials). We identified candidate FnrN/FixK-binding sites upstream of such genes not only in *B. japonicum*, but also in several other rhizobia genomes. Nitrate reductase (*napEDABC*) operon has FnrN/FixK-binding sites in *Bradyrhizobium* spp., *Rhizobium sp.* NGR234, and *S. meliloti*, genes for nitrous oxide reductase (*nos*), in *Bradyrhizobium* spp. and *S. meliloti*, and genes for nitrite reductase (*nirKV*), in nine *Rhizobiales* genomes.

In *Rhizobiales*, the FnrN/FixK-regulatory network becomes more subdivided as more genes for transcription factors turn out to be under their control. These transcription factors include not only FnrN/FixK themselves and FixLJ, but Crp-Fnr superfamily transcription factor AadR, NO respiration regulator NosR, and some uncharacterized transcription regulators from the Crp-Fnr superfamily.

We observed that FnrN/FixK might control the *phbC* gene encoding poly-beta-hydroxybutyrate (PBH) polymerase in some *Rhizobiales*. It was shown previously that *phbC* mutants of *S. meliloti* resulted in nodule formation delay in *Medicago truncatula* and young *M. sativa* nodules (Wang et al., 2007). PHB synthesis genes are downregulated in *M. huakuii* 7653R during symbiosis (Peng et al., 2014). PBH synthesis and accumulation was experimentally studied in nitrogen-fixing Gammaproteobacteria *Azotobacter vinelandii* and was shown to be involved in encystment (Stevenson and Socolofsky, 1966). A possible link between the PHB metabolism and nitrogen fixation has been suggested previously (Oelze, 2000), as in low oxygen concentration redundant carbon is stored as PHB rather than is dissimilated to provide reducing equivalents and energy for nitrogen fixation.

## Discussion

### Varying Complexity of Nitrogen-Fixation Regulatory Cascades

The analyzed genomes of *Alphaproteobacteria* demonstrate considerable variability in the repertoire of studied regulators and the composition of their regulons. Some nitrogen-fixing bacteria possess simple networks consisting of two independent regulons, nitrogen fixation genes under the NifA control and the genes involved in the adaptation to oxygen concentration under the FnrN control. These organisms include *Rhodospirillales, R. sphaeroides*, and *Z. mobilis*. Other Alphaproteobacteria have a larger repertoire of transcription factors and more complicated, interdependent cascades (Supplementary Figure S3 in the Supplementary Materials).

**FIGURE 4:**
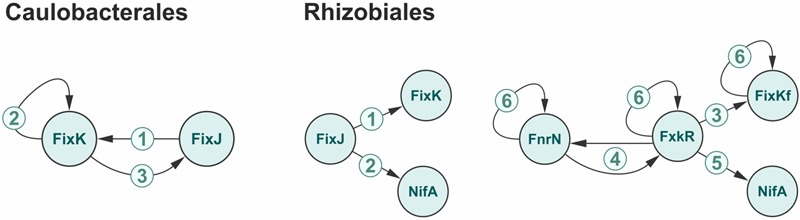
**Gradual growth of complexity for the nitrogen regulatory network.** The numbers represent the levels of the network complexity. For the details see the text.

While members of *Caulobacteriales* do not fix nitrogen but contain orthologs of FixK/FnrN-like transcription factors regulating nitrogen fixation in other bacteria, indicating that the role of these regulatory systems is not so much in nitrogen fixation, as in adjustment of redox processes. All four *Caulobacteriales* species have the FixLJ system and FixK, and the following levels of the network complexity can be observed (**Figure 4**; Supplementary Figure S3 in the Supplementary Materials): (1) FixLJ controlling the *fixK* gene as in all studied *Caulobacteriales*); (2) additional autoregulation of *fixK* regulates its own gene as in *Phenylobacterium zucineum, Caulobacter crescentus*, and *C. segnis*); (3) further, FixK controlling *fixJ*, hence closing the feedback loop as in *C*. *crescentus, C. segnis, and Caulobacter* sp. K31).

The most complex regulatory network functions in *Rhizobiales* (**Figure 4**; Supplementary Figure S3 in the Supplementary Materials): (1) FixLJ is always on the top of the cascades and in all nitrogen-fixing bacteria it controls the *fixK* gene (Agron et al., 1993; Fischer, 1994; Robles et al., 2006). In the simplest case NifA controls nitrogen-fixation genes independently (*B. japonicum, B*. sp. BTAi, *R. palustris*). (2) In some bacteria (*S. meliloti, M. loti, A. caulinodans, Xanthobacter autotrophicus*) FixLJ controls the *nifA* and hence establishes a link between the nitrogen fixation process and oxygen concentration (Kaminski and Elmerich, 1991; Fischer, 1994). (3) An alternative two-component system, hFixL-FxkR may co-exist with FixLJ (*S. meliloti*) or substitute it (*R. leguminosarum, R. etli*). Analogously to FixLJ controlling *fixK*, this alternative hFixL-FxkR system controls the *fixKf* gene (Zamorano-Sanchez et al., 2012). (4) In the organisms with the alternative hFixL-FxkR two-component system (*R. etli* and *R. leguminosarum*), this system forms a loop with FnrN (Boesten and Priefer, 2004). (5) In *R. leguminosarum*, hFixL-FxkR controls the *nifA* gene, *cf*. case (2), (6) Additionally, in some *Rhizobiales* several transcription factors regulate their own genes (Batut et al., 1989; Kaminski and Elmerich, 1998; Nellen-Anthamatten et al., 1998; Girard et al., 2000; Lopez et al., 2001; Martinez et al., 2004; Nukui et al., 2006; Dufour et al., 2010).

The comparative-genomics based approach used here allowed us to identify some novel transcriptional regulators in analyzed genomes, namely RPA1090 (Crp-Fnr family), CydR (ArsR family), and Bll2758 (OmpR family), based on their co-localization with nitrogen-fixation genes and/or regulation by nitrogen-fixation factors. However, each of these regulators was found in a small number of closely related genomes, precluding further comparative-genomic analysis of the corresponding regulons. However, we can distinguish between functionally significant conservancy, i.e., conservancy of gene context and/or predicted binding sites in a variety of genomes, from the overall conservancy caused by mere taxonomic proximity of the analyzed genomes. We expect that more sequenced genomes will make it possible to characterize binding motifs and cognate regulons for these regulators. Anyway, the narrow distribution of these regulators seems to point to their minor roles in the regulatory networks. Thus, we believe that the main observations made here are sufficiently robust.

### Evolution of Nitrogen Fixation-Related Regulatory Network: Major Events

**FIGURE 5:**
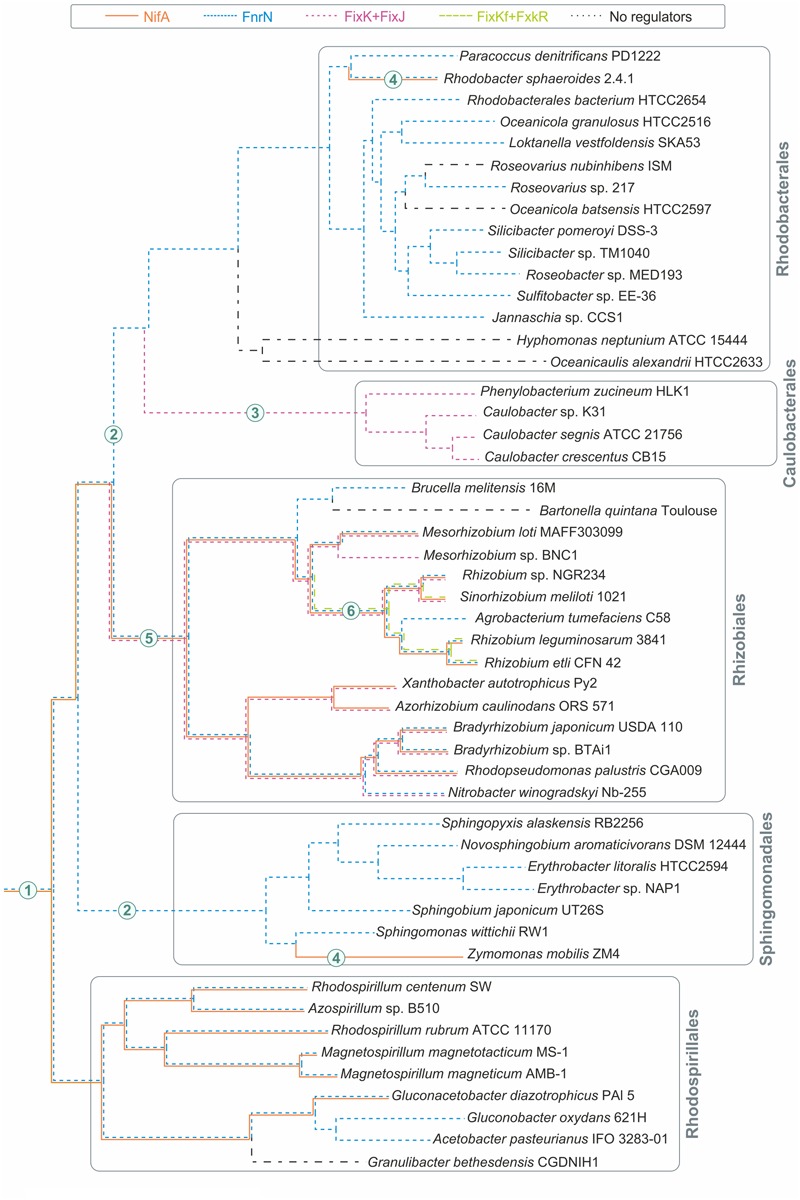
**Major evolutionary events in analyzed *Alphaproteobacteria*.** The species tree was extracted from the MicrobesOnline web-resource (Dehal et al., 2010). The numbers on the branches represent major events: (1) NifA is a regulator of nitrogen fixation, FnrN is a regulator of aerobic respiration; (2) loss of NifA; (3) loss of FnrN, horizontal transfer of the *fixLJ-fixK* gene cluster from *Rhizobiales*, and expansion of the FixK regulon by the *cyd* and *cco* genes; (4) horizontal transfer of the nitrogen fixation locus from *Rhodospirillales*; (5) appearance of FixK and FixJ; (6) appearance of FixKf and FxkR.

Based on our results and the species tree for the analyzed genomes, we attempted to reconstruct the evolutionary history of the analyzed regulons (**Figure 5**). Here we used the species tree extracted from the MicrobesOnline database. The species tree is based on concatenated protein sequences for 31 proteins universally present as single copies in complete bacterial genomes (Wu and Eisen, 2008). The evolutionary history of the analyzed regulons features numerous gains, losses, and horizontal gene transfers.

Almost all analyzed taxa (except *Caulobacteriales*) contain representatives that possess orthologs of FnrN, so we propose that FnrN has been present in the last common ancestor of *Alphaproteobacteria*. Subsequently, it was lost in *Caulobacteriales* and substituted by the oxygen-sensing FixLJ system and the FixK regulator (**Figure 5**). The phylogenetic analysis of *Caulobacteriales* proteins for the FixLJ-FixK regulatory cascade placed these proteins within the *Rhizobiales* branch (**Figure 1**; Supplementary Figure S4 in the Supplementary Materials). Thus, the loss of the *fnrN* gene in *Caulobacteriales* was accompanied by horizontal transfer of the *fixLJK* gene cluster from a *Rhizobiales* organism. Most probably, the transfer of the *fixLJK* cluster preceded the loss of FnrN, and the expansion of the FixK regulon was due to binding of transferred FixK to existing FnrN-binding sites. This explains the similarity of the FixK regulon in *Caulobacteriales* and the FnrN regulon in *Rhodobacterales*, which are closely related to *Caulobacteriales*.

NifA orthologs are widespread in two distant branches (*Rhizobiales* and *Rhodospirillales*) and, hence also might be present in the common *Alphaproteobacterial* ancestor. *Sphingomonadales* and *Rhodobacterales* have single known members with NifA orthologs suggestive of horizontal gene transfer. The phylogenetic analysis of the NifA proteins (Supplementary Figure S5 in the Supplementary Materials) shows that NifA of *Z. mobilis* does not form a separate taxon-specific branch but is located within *Rhodospirillales*, clustering with the NifA protein from *G. diazotrophicus* Pal5. All NifA-regulated genes in *Z. mobilis* genome form a single chromosomal cluster (*ZMO1808-37*). This cluster includes the *nifA* gene itself, nitrogenase operon *nifHDKENX-fdxB-nifQ*, operons *nifB-fdxN* and *iscN-nifUSVW-modD* for nitrogenase maturation, and operon *rnfABCDGEH* for the electron transport complex. In *G. diazotrophicus*, NifA-regulated genes form an almost identical gene cluster, the only difference being the absence of the *rnf* operon in *G. diazotrophicus* and the absence of the *fixBAC* genes in *Z. mobilis*. The phylogenetic analysis of NifA-regulated genes (only proteins of at least 200 amino acids were analyzed) demonstrated that the Nif and ModD proteins of *Z. mobilis* are clustered on the trees with the corresponding proteins from *Rhodospirillales* (Supplementary Figures S5B–J, in the Supplementary Materials), as well as *modD* and *nif* genes tends to form a single chromosomal cluster in analyzed *Rhodospirillales* genomes. Unfortunately, the *rnf* operon is present in only four analyzed genomes and its phylogenetical analysis is irrelevant. Thus, based on the phylogeny of NifA, we propose that *Z. mobilis* obtained NifA together with operons *nifHDKENX-fdxB-nifQ, nifB-fdxN*, and *iscN-nifUSVW-modD* via horizontal transfer from a *Rhodospirillales* organism. Regarding the *rnf* operon, two alternative scenarios are possible, horizontal transfer together with NifA and other regulated genes, and expansion of the NifA regulon to the *rnf* operon in *Z. mobilis* after horizontal transfer.

*Rhodobacterales, R. sphaeroides* might have gained *nif* gene cluster in a similar manner. In the phylogenetic tree, NifA from *R. sphaeroides* belongs to the *Rhodospirillales* branch clustering with NifA from *Rhodospirillum centenum* SW (Supplementary Figure S5A in the Supplementary Materials). In *R. sphaeroides*, the *nifA* gene is co-localized with NifA-regulated *nif* genes for nitrogen fixation (*RSP_0532-46*), whereas three other NifA-regulated operons, *rnf* (*RSP_3192-98*), *modD* (*RSP_2501*), and *RSP_2791-90*, are located separately. In *Rhodospirillales*, the *nif, rnf*, and *modD* genes, but not *RSP_2791-90*, are also members of the NifA regulon. Phylogenetic analysis clusters most Nif proteins and ModD from *R. sphaeroides* with the proteins from *Rhodospirillales* (Supplementary Figures S5B–J, in the Supplementary Materials). Thus, we propose that the *nif-modD* cluster was transferred to *R. sphaeroides* from a *Rhodospirillales* genome, then the *modD* gene was re-located, and the NifA regulon expanded by the *RSP_2791-90* operon. As for the *rnf* operon, both scenarios, horizontal transfer and regulon expansion, are possible, as in *Z. mobilis* (see above).

The evolutionary history of *Rhizobiales* is the most complicated among the studied taxa (**Figure 5**). The main event here might be recruitment to the network of the FixK protein and the FixLJ system in the common ancestor of *Rhizobiales*. FixK appears to be the result of duplication of the ancestral *fnrN* gene with subsequent loss of the iron-sulfur cluster. FixLJ might result from recruitment of an existing two-component system. An additional regulatory cascade, hFixL-FxkR-FixKf, appeared in the common ancestor of *Rhizobiaceae*, as seen now in *Agrobacterium tumefaciens, Rhizobium* spp., and *S. meliloti*. FixKf appears to be a paralog of *fnrN* resulting from duplication in the common ancestor of *Rhizobiaceae* while the subsequent hFixL-FxkR system was recruited later.

### Why So Redundant?

The response to molecular oxygen in *Alphaproteobacteria* is characterized by a seeming redundancy of regulators. Firstly, two different systems are used for oxygen sensing, FnrN, which senses oxygen directly via the iron–sulfur cluster, and regulatory cascades, FixLJ-FixK or hFixL-FxkR-FixKf. Secondly, in eight studied genomes these systems are present in multiple copies. For example, five copies of *fixK* gene were found in *Bradyrhizobium* sp. BTAi1 (Supplementary Table S3 in the Supplementary Materials). Based on the literature analysis (for references see below) and results of this study we suggest the following explanation for this redundancy.

The need for two systems, FnrN and FixLJ-FixK, may be explained by different subcellular localization of oxygen-sensing structures in these systems. At that, FnrN senses intracellular oxygen, whereas FixL/hFixL senses extracellular oxygen. Absence of extracellular oxygen indicates that bacterium is inside a nodule and the transcriptional profile should be adapted to this new environment. Additionally, regulation via a cascade yields large response time in comparison to regulation by a single one-component regulator. Firstly, activation by two-component regulatory system takes at least 75 s by itself (Gao and Stock, 2015). Secondly, in the case of regulatory cascade, this activation should be followed by transcription and translation of activated gene. The speed of RNA elongation during transcription in Bacteria is of about 40–80 nucleotides per second (Bremer and Yuan, 1968; Vogel and Jensen, 1994), whereas a maximal speed of translational elongation is 20 amino acids per second (Dennis and Bremer, 1974; Dennis and Nomura, 1974; Young and Bremer, 1976). The length of the shortest FixK protein analyzed in this work, BBta_7767 from *Bradirhizobium* sp. BTAi1, is 153 amino acids. Thus, even if translation is coupled with transcription and both these processes are performed at a maximal speed, biosynthesis of even the shortest regulatory protein takes between 7 and 8 s. Taking into account that all other FixK proteins are longer, initiation of transcription and translation also should take time, and that transcription and translation coupling is relatively rare (Bakshi et al., 2012), we can be sure that real time of the response through this regulatory cascade is longer than the response resulting from regulation by a one-component regulator. Hence, FnrN is used for fast response to the concentration of intracellular oxygen, whereas FixLJ-FixK cascades are used for slower and long-term response to the concentration of extracellular oxygen. Additionally, at least in some *Rhizobiales*, the FixLJ two-component systems provide a link between oxygen sensing and nitrogen fixation. Thus, the *nifA* gene is regulated by FixJ in *S. meliloti, M. loti*, and *A. caulinodans*, and by FxkR in *R. leguminosarum*. Thus, the presence of two alternative systems, the FnrN and FixLJ-FixK cascades, is not a redundancy, but is necessary for fine-tuning of the oxygen response in changing environment.

The presence of multiple copies of regulators can be explained by two different, but not mutually exclusive hypotheses. The first one is that the presence of multiple copies provides protection against the regulator destruction. As the oxygen response plays a crucial role in nitrogen fixation, destruction of an oxygen-responsive transcription regulator may be extremely harmful. So, multiple oxygen-responsive regulators serve as backups. The second hypothesis is that different paralogs have different affinities to cognate binding sites in a genome. Binding of paralogous transcription regulators to the same binding sites with different affinities has been previously demonstrated for pairs of regulators from *Escherichia coli*, such as GalS/GalR (Geanacopoulos and Adhya, 1997), ExuR/UxuR (Suvorova et al., 2011), and NarL/NarP (Darwin et al., 1997). Similarly, copies of FnrN proteins may have different sensitivity to oxygen. Hence, they can differently change transcription levels of the regulated genes in response to varying levels of oxygen. Under this explanation, multiple copies of regulators form a mechanism for fine-tuning of the oxygen response.

## Author Contributions

DR and MG conceived and designed the research project. DR, OT, and JČ performed the comparative genomic analysis to reconstruct regulons. DR, OT, and MG wrote the manuscript. All authors read and approved the final manuscript.

## Conflict of Interest Statement

The authors declare that the research was conducted in the absence of any commercial or financial relationships that could be construed as a potential conflict of interest.

## Footnotes

1 http://www.ebi.ac.uk/Tools/msa/muscle
