# Supplementary material for: Nitrogen Fixation and Molecular Oxygen: Comparative Genomic Reconstruction of Transcription Regulation in Alphaproteobacteria
Source: Front Microbiol. 2016 Aug 26;7:1343. doi: 10.3389/fmicb.2016.01343 (PMC4999443; doi:10.3389/fmicb.2016.01343)
Supplement: Supplementary file 2 [file Table_2.PDF]

Table S2. **References for web-pages of the reconstructed regulons at RegPrecise database.**

**Taxa**	**Transcriptional Regulator(s)**	**Reference**
Caulobacterales	FixK	http://regprecise.lbl.gov/RegPrecise/regulog.jsp?regulog_id=6531
	FixJ	http://regprecise.lbl.gov/RegPrecise/regulog.jsp?regulog_id=6527
Rhizobiales	NifA	http://regprecise.lbl.gov/RegPrecise/regulog.jsp?regulog_id=6581
	FixK/FnrN/FixKf/AadR	http://regprecise.lbl.gov/RegPrecise/regulog.jsp?regulog_id=6503
	FixJ	http://regprecise.lbl.gov/RegPrecise/regulog.jsp?regulog_id=6526
	FxkR	http://regprecise.lbl.gov/RegPrecise/regulog.jsp?regulog_id=6580
Rhodobacterales	NifA	http://regprecise.lbl.gov/RegPrecise/regulog.jsp?regulog_id=6583
	FnrN	http://regprecise.lbl.gov/RegPrecise/regulog.jsp?regulog_id=6530
Rhodospirillales	NifA	http://regprecise.lbl.gov/RegPrecise/regulog.jsp?regulog_id=6584
	FnrN	http://regprecise.lbl.gov/RegPrecise/regulog.jsp?regulog_id=6532
Sphingomonadales	NifA	http://regprecise.lbl.gov/RegPrecise/regulog.jsp?regulog_id=6582
	FnrN	http://regprecise.lbl.gov/RegPrecise/regulog.jsp?regulog_id=6529
