# Supplementary material for: Nitrogen Fixation and Molecular Oxygen: Comparative Genomic Reconstruction of Transcription Regulation in Alphaproteobacteria
Source: Front Microbiol. 2016 Aug 26;7:1343. doi: 10.3389/fmicb.2016.01343 (PMC4999443; doi:10.3389/fmicb.2016.01343)
Supplement: Supplementary file 5 [file Image_1.PDF]

Figure S1. **Multiple alignment for FnrN/FixK-like proteins.** Cysteine residues required for formation of iron-sulphur cluster are labelled by light-green background. HTH domain residues undergoing specific interaction with DNA nucleotides are labelled by orange background.

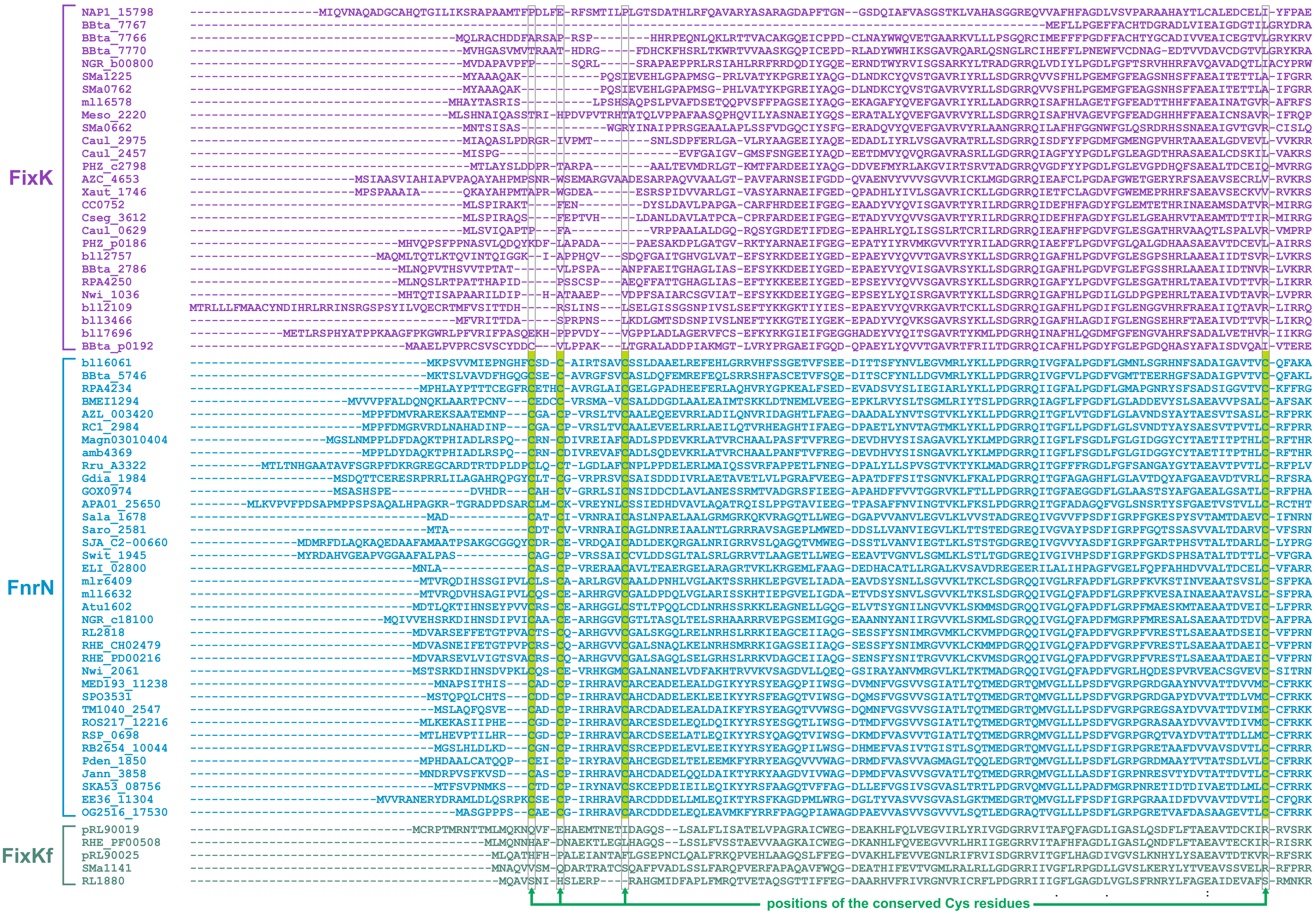

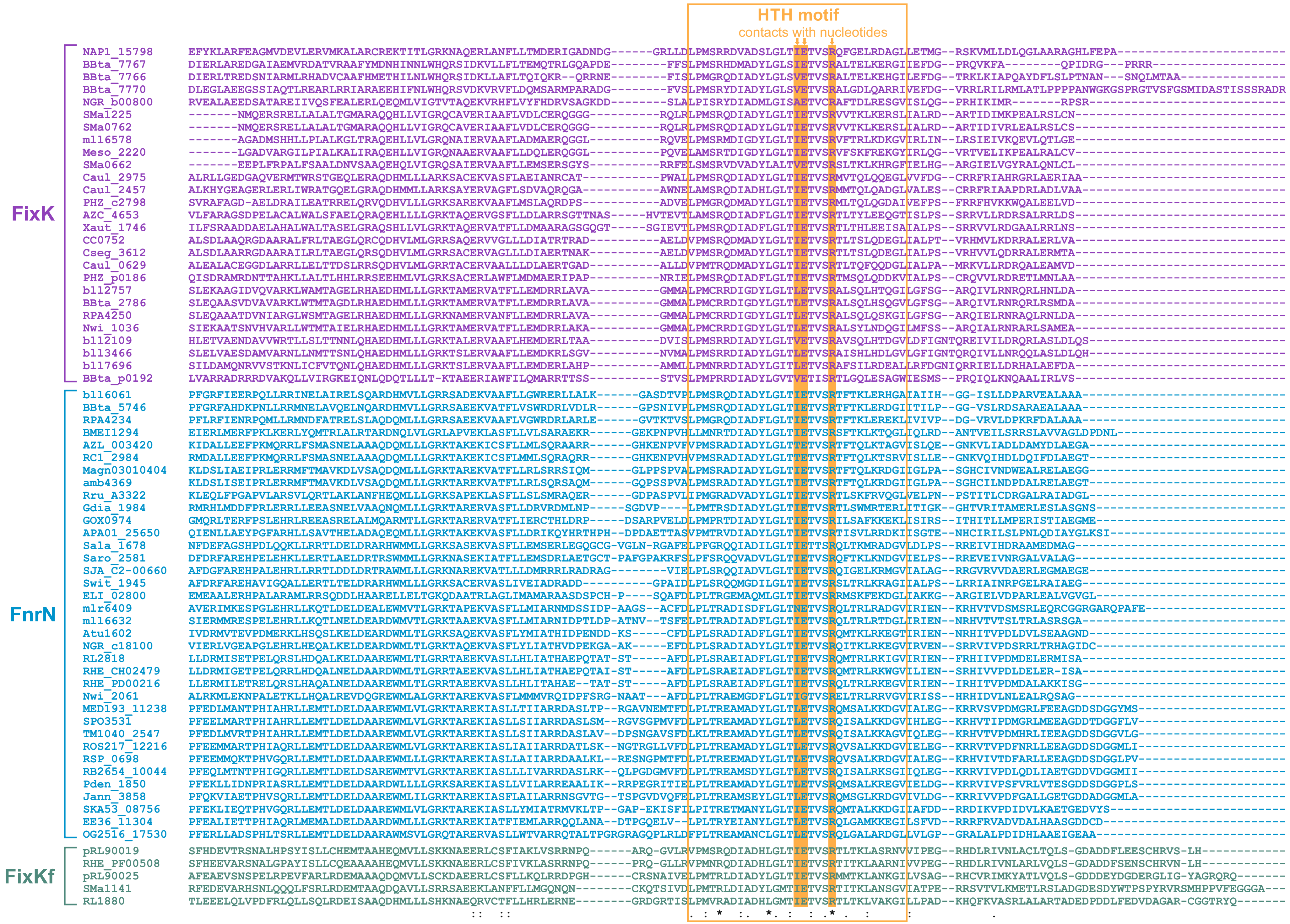

HTH motif
contacts with nucleotides
FixK
NAP1_15798
BBta_7767
BBta_7766
BBta_7770
NGR_b00800
SMa1225
SMa0762
mll6578
Meso_2220
SMa0662
Caul_2975
Caul_2457
PHZ_c2798
AZC_4653
Xaut_1746
CC0752
Cseg_3612
Caul_0629
PHZ_p0186
bll2757
BBta_2786
RPA4250
Nwi_1036
bll2109
bll3466
bll7696
BBta_p0192
FnrN
bll6061
BBta_5746
RPA4234
BMEI1294
AZL_003420
RC1_2984
Magn03010404
amb4369
Rru_A3322
Gdia_1984
GOX0974
APA01_25650
Sala_1678
Saro_2581
SJA_C2-00660
Swit_1945
ELI_02800
mlr6409
mll6632
Atu1602
NGR_c18100
RL2818
RHE_CH02479
RHE_PD00216
Nwi_2061
MED193_11238
SPO3531
TM1040_2547
ROS217_12216
RSP_0698
RB2654_10044
Pden_1850
Jann_3858
SKA53_08756
EE36_11304
OG2516_17530
FixKf
pRL90019
RHE_PF00508
pRL90025
SMa1141
RL1880
