# Supplementary material for: Nitrogen Fixation and Molecular Oxygen: Comparative Genomic Reconstruction of Transcription Regulation in Alphaproteobacteria
Source: Front Microbiol. 2016 Aug 26;7:1343. doi: 10.3389/fmicb.2016.01343 (PMC4999443; doi:10.3389/fmicb.2016.01343)
Supplement: Supplementary file 6 [file Image_2.PDF]

Figure S2. **Multiple alignments for upstream regions of *fixK* and *fixKf* genes from Rhizobiales and Caulobacterales.** Gene coding region are shown in bold, predicted FixJ and FixJf binding sites are shown in bold underlined blue font.

fixK Rhizobiales (FixJ binding site)

```
AZC_4653     GTAAA----ATCA-------------------------------------------------------------------------
Xaut_1746    AACATCTTTGCGACCCTGCCCCTTCAGC---------------------------------------------------------C
PHZ_c2798    GAAAG----TCGATCTCCTTGCTTCGCCGGCAGCTCTAGCGGGGCAGTGCCGCGGCGGATGTGACGGCGATCAAAGCGCCCCCTGC
NWI_1036     GAACA----TTCA-------------------------------------------------------------------------
RPA4250      GCAGACTCTCTCGCCCCTTATCTTAAGATCCTGTTAAGACATCGGTTTTCCCCACGGAAGCGGACGAGGGCTCACGCCCCGAG---
bll2757      GA------------------------------------------------------------------------------------
BBta_2786    GAGCATTCGACTACCGCCCAGC-----------------------------------------------------GCGCCTCCTGA


AZC_4653     --------CCTACGCAATACTACTTAAGGGCTGGGCCCTAAGGAGTGAATCCGAATTTTTGCAGGGCGGTCAACGGGCTATCCCTT
Xaut_1746    GGTTCCGGCCTCCGTAAAACTACTTAAGGGCACCGACTTAAGTGTTCGATCCGAATTTTGCGATGCGTCAATGCCGGTTATCCCTT
PHZ_c2798    GGGAC---CCTCCGTAGAACTACTTAGGTGCGACCCCTTAGGGGGACGCCACGAATTTCTTGCGGCCCGGCGCAGGCGTACCCCTT
NWI_1036     --------CCTCCGTAAGACCCCTTAGGAGGCTGCCCTTAGGATATCGACCGAAATACGCCTGCGCGCCCGCATTTGGTACACGA-
RPA4250      --------CCTACGTAGACCTACTTAGGGGAACCCACTTAAGATATGGAGCGAAATACCTCGTCCTCGGAATCTTCCGTACAACCC
bll2757      --------CCTACGGGGTTCTACGTAAGGCACCCCCCTTAAGATATCGCTCGAAATTTTCGAACCTCCCGATACCGCGTACCAATG
BBta_2786    GGGGCCGATCTACGTATATCTACGTAAGGGAACGTCCTTAAGATAACGGCCGAAATGTCCTTAACGGGAGCCGCCGTGTACCACAT
                      ** **     *  * ** *        * ** *           ***                      **

AZC_4653     -CATCAACGAAATT--CGGAGATGAGACCGATGTCGATC------------------GCCGCCAGCGTCATCGCCCATATCGCTCC
Xaut_1746    TCTGCAACGTGATGGAAGGGTAAACC---GATGCCGAGCC------------------CCGCTGCAGCGATCGCCCAGAAGGCTTA
PHZ_c2798    -CGTCATC---------------------GACGCAGGAAC-----------------TTCGCCATGACCCTCGCCTACTCGCTGGA
NWI_1036     -AGCCATCCGCATCGA--GAGATGGC-GACATGCACACCCAGACGATCTCCGCTCCGGCCGCCAGGATTCTCG---ACATTCCGCA
RPA4250      TGGCCATCCG-ATCAAAGGAGATGGC-CAGATGCTGAACCAGTCGCTCAGGACGC---CCGCCACCACCCACGCCCCCATCG----
bll2757      -CGTCATCA----CAACGGAGATGGCGCAGATGCTGACCCAGACACTCAAGACCCAGGT------GATCAACACCCAAATCGGTGG
BBta_2786    -CGCCATCCGCCGCAA-GGAGATGGT---CATGCTCAATCAGCCGGTTACACATTCGGTCGTCACCCCCACCGCCACCGT------
                 ** *                      * *                                      *
```

fixK Caulobacterales (FixJ binding site)

```
Caul_0629    TAGGCGCGAGATCTCGGCGCTCCCAG----GTCGAGGACGGGCATTTCGTGCCTAGGTATTCGC-CCTTAGGCGTCGGTCCCGACT
Cseg_3612    TCCCGGTCAAA-ACAGGTTTGTAAAGGGGCGGCGCATAAGGGATTTCC---CTTAGCGAGGC-CTCCTTAGGCGGCCCCCCCGAAT
CC0752       TCGGCGTCAAA-ATCGCCGCCCTCAGCATCGCCGCCTAAGGGACTCCC---CTTAGCGGACCGCTCCTTAGGCGCATGCCCCGAAT
             *    *  * *    *        **    * **   * ***  *  *   * ***     * * *********     ***** *

Caul_0629    ---GTACCGCTTGGGCTCCGCGGACGAGACTGCGGGCCGAAGACGAGACCGGAGACCGCCATGCTGTCCGTGATTCAAGCGCCCAC
Cseg_3612    TTCGCGAC-TTTTGCTTTGGCGCAATAACCCTCGGGC---AACTG-----GGGGTTTCCAATGCTGTCTCCGATCC--GCGCTCAA
CC0752       TTCGAACCCGTTTGAGATGGCGCAAAAAACCCACAAC---AAATGAG---GGGGTTTCCCATGCTGTCTCCGATCC--GCGCCAAG
                *   *  ** *     *** *  *  *      *   *   *     ** *    * ********   *** *  ****  *
```

fixKf Rhizobiales (FxkR binding site)

```
SMa1141       -------GCAAGGTCGGTGCATTCGCCATGAGTAACCCCAATGCCGCTGTTCCACTC----------------------------
RHE_PF00508   ATTTCGAGGCAGGACTGTCATGAAGCCGGGAATGGCTCC-------TTGATCTACATCAACAAGGGGCCTGGAAGCGTCGGACGA
pRL90019      -------GCCAGGACTGTCACGAAGCCGCGAATTGCTCC-------TTGATCTACATCAACAAGAGGCCAGCAAG-ACGGGGCGA
                     *  *** **      ***  ** *  * **        ** ** **

SMa1141       -CCAGCGCCGTTACACTCTGTTACAATATCGTACCCATTTTTGCACTCGTGTCGTCATGGGATTACGGCCTGGCGTTCAACTCTT
RHE_PF00508   ACCGTCGCTGTTACACACTGTTACAAACTTTCACCCTTTGGCACCGTCCGCTCATTCCGGTGTGACGCAGCGGCCTTCTACTGCA
pRL90019      ACCGCCGCGGTTACAAACTGTTACAAACTTTCACCGGTTAGCACTGTCCGATCATTCCGCTGTGACGCAGAGGCGTTCCAATGTG
               **  *** ******  *********  *   ***  **    *  **   ** *   *   * ***    *** *** * *

SMa1141       TCCATCAGCAAAGATGGA---GGGGACGCTCAT---GAACGCGCAGGTGGTT-AGCATGCAGGATGCTCGCACAAGAGCAACGTG
RHE_PF00508   CCCACTGCCACAAGGGGACACGACGATGCTGATGCAGAACAACCACGCGTTCGACAATGCCGAG---------------AAGACG
pRL90019      CCGACCGACAATGAGGAACACGACGATGCTGATGCAGAAAAACCAGGTGTTCGAACATGCCGAA---------------ATGACG
               * *    **     * *   *  ** *** **   ***    ** * * *  *  **** *                 *    *
```
