# Supplementary figures and images for: Nitrogen Fixation and Molecular Oxygen: Comparative Genomic Reconstruction of Transcription Regulation in Alphaproteobacteria
Source: Front Microbiol. 2016 Aug 26;7:1343. doi: 10.3389/fmicb.2016.01343 (PMC4999443; doi:10.3389/fmicb.2016.01343)

Figure S3. Interconnectivity in reconstructed regulons.

Caulobacterales

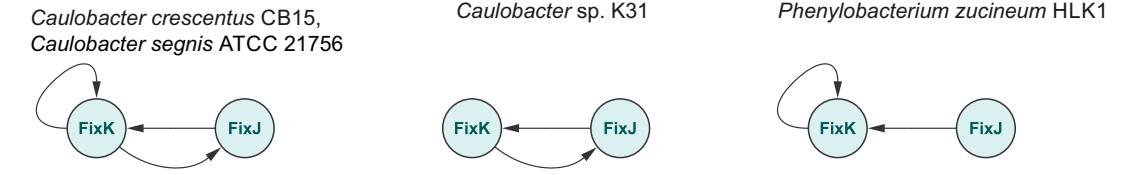

Rhizobiales

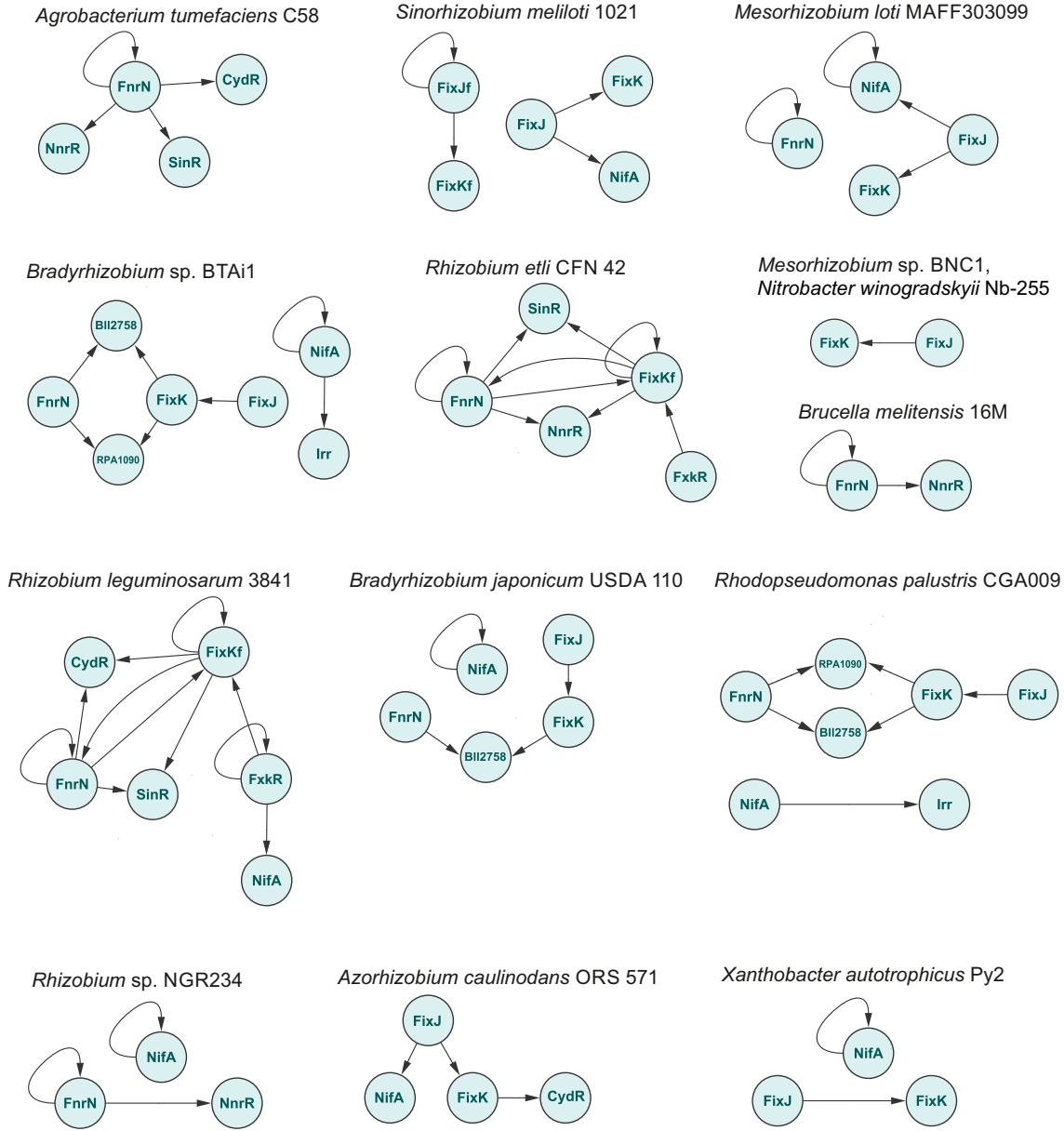

Supplement: Supplementary file 7 [file Image_3.PDF]

Figure S4. Maximum likelihood phylogenetic trees for (A) FixJ/FxkR and (B) FixL/hFixL

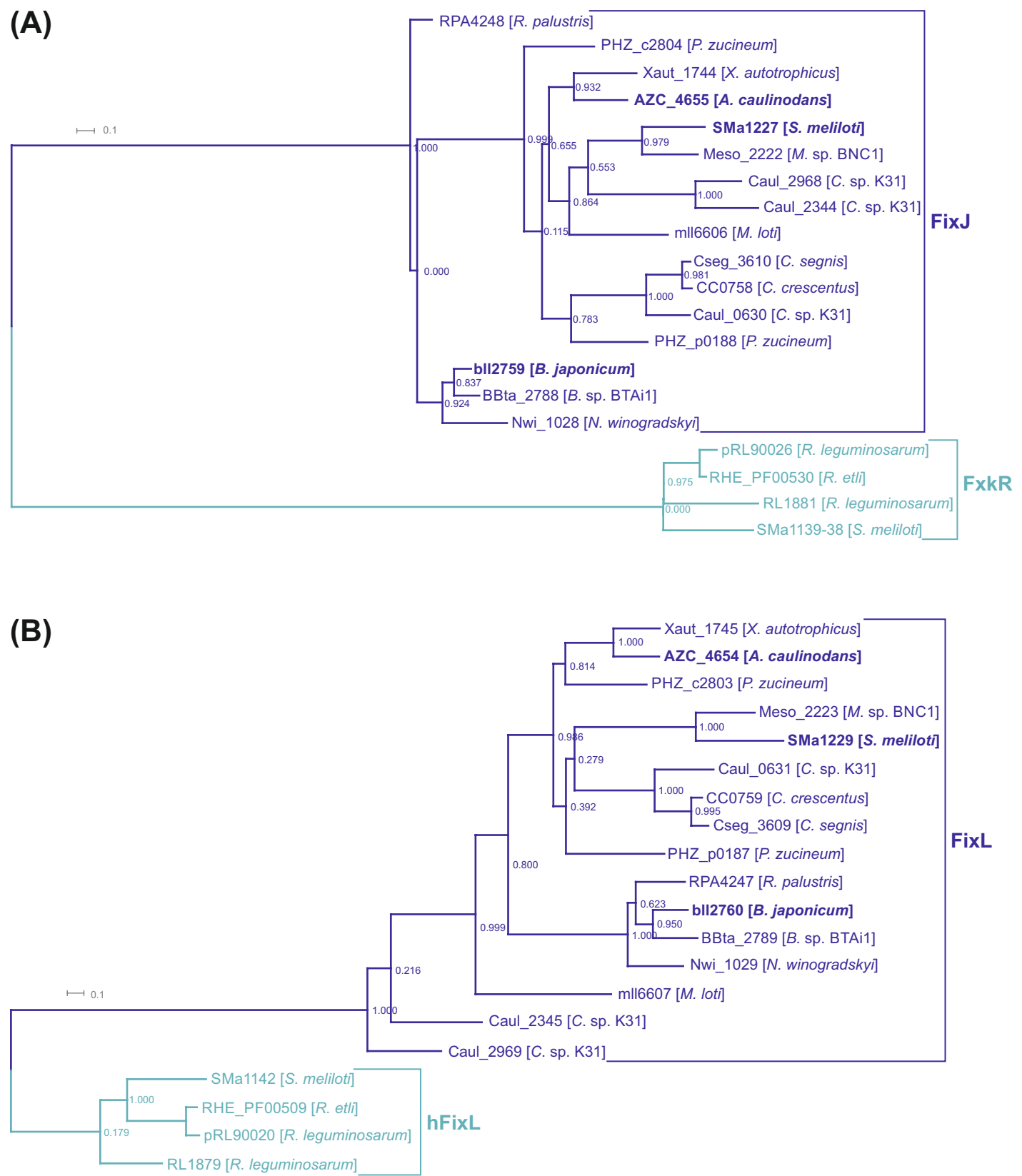

Supplement: Supplementary file 8 [file Image_4.PDF]
