# Supplementary material for: Nitrogen Fixation and Molecular Oxygen: Comparative Genomic Reconstruction of Transcription Regulation in Alphaproteobacteria
Source: Front Microbiol. 2016 Aug 26;7:1343. doi: 10.3389/fmicb.2016.01343 (PMC4999443; doi:10.3389/fmicb.2016.01343)

Figure S5. **Maximum likelihood phylogenetic tree for NifA proteins.**
All the trees were mid-point rooted. (A) NifA, (B) NifB, (C) NifD, (D) NifE, (E) NifH, (F) NifK, (G) NifN, (H) NifS, (I) NifV, (J) ModD, (K) CydA, (L) CydB, (M) CydC, (N) CydD.

(A) **NifA**

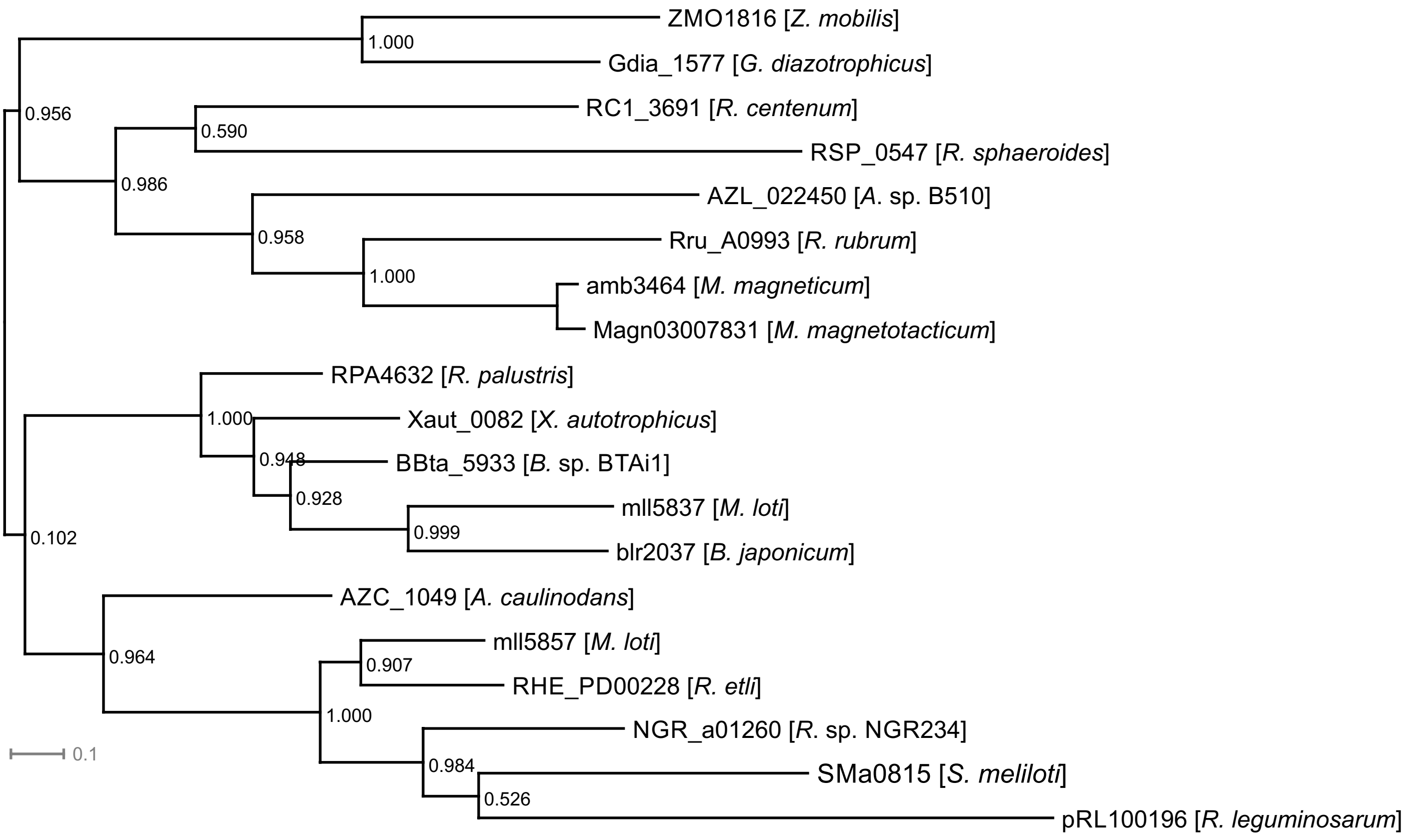

(B) **NifB**

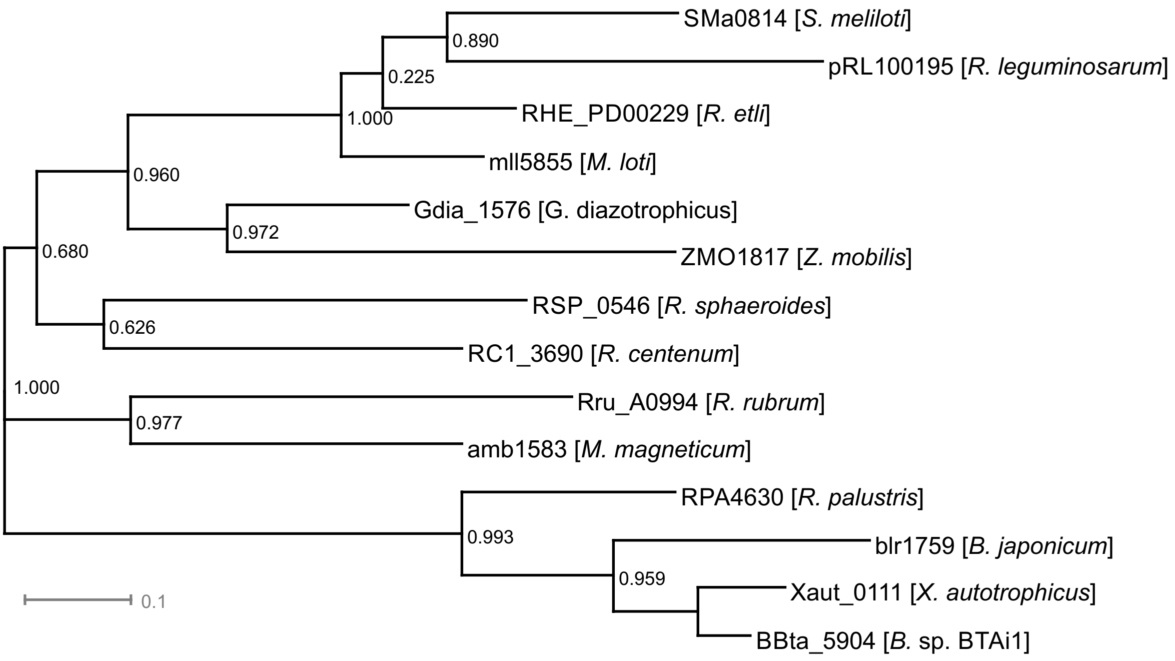

(C) **NifD**

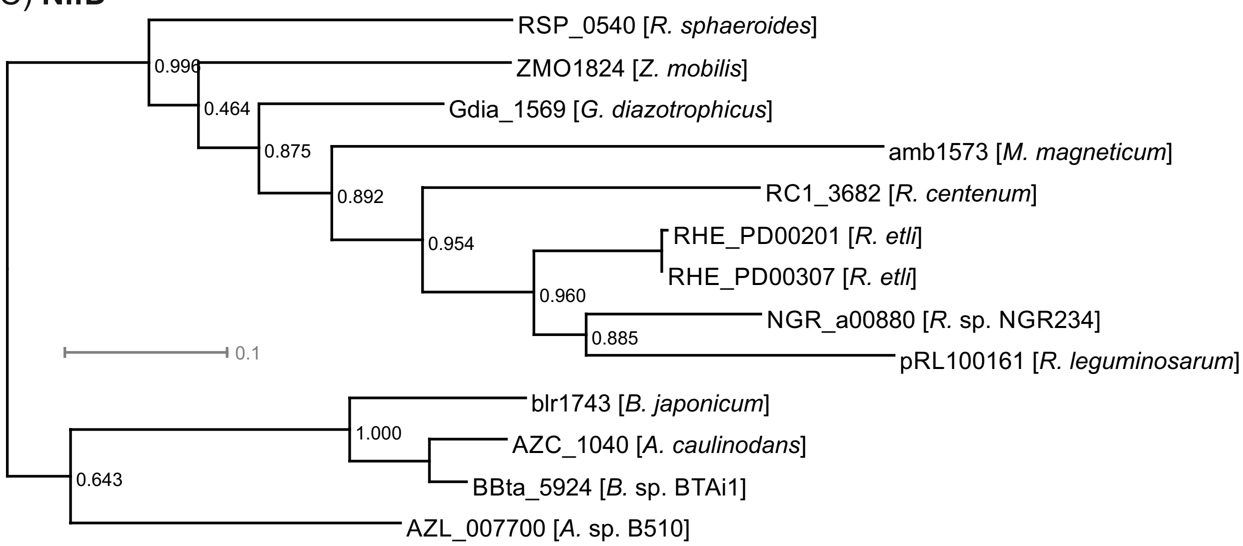

(D) **NifE**

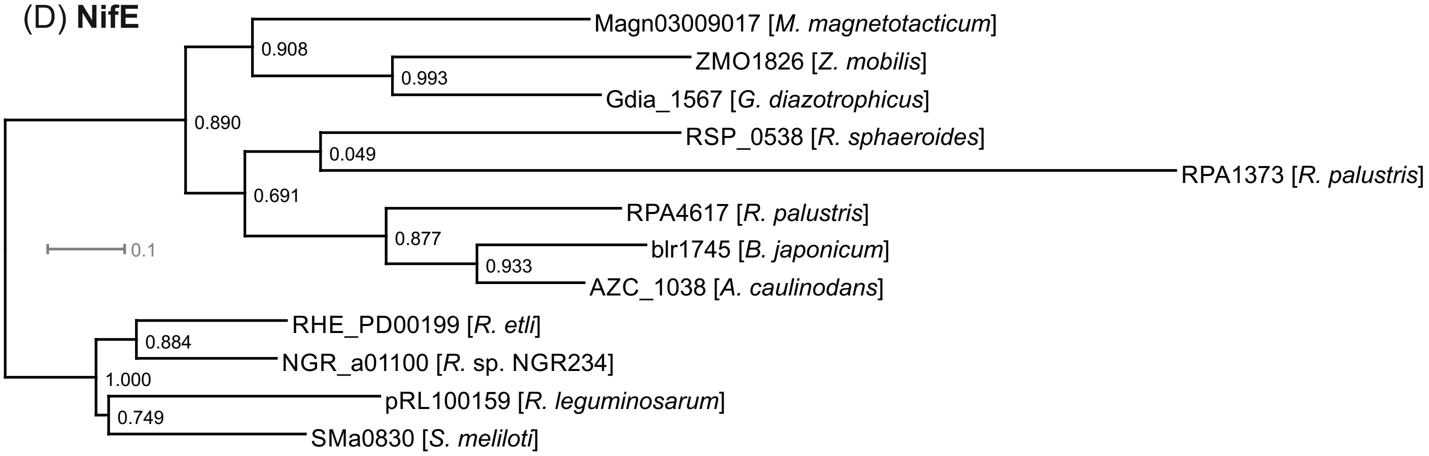

(E) **NifH**

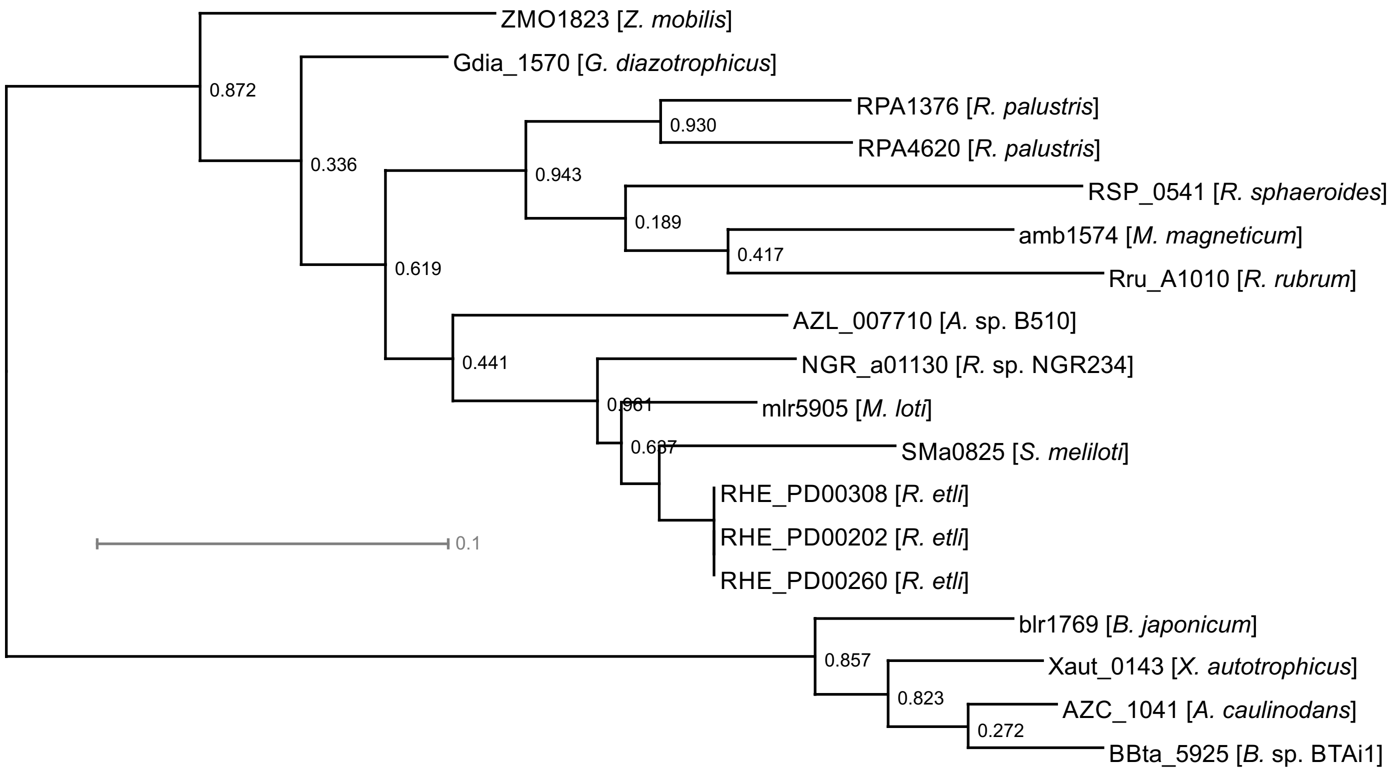

(F) **NifK**

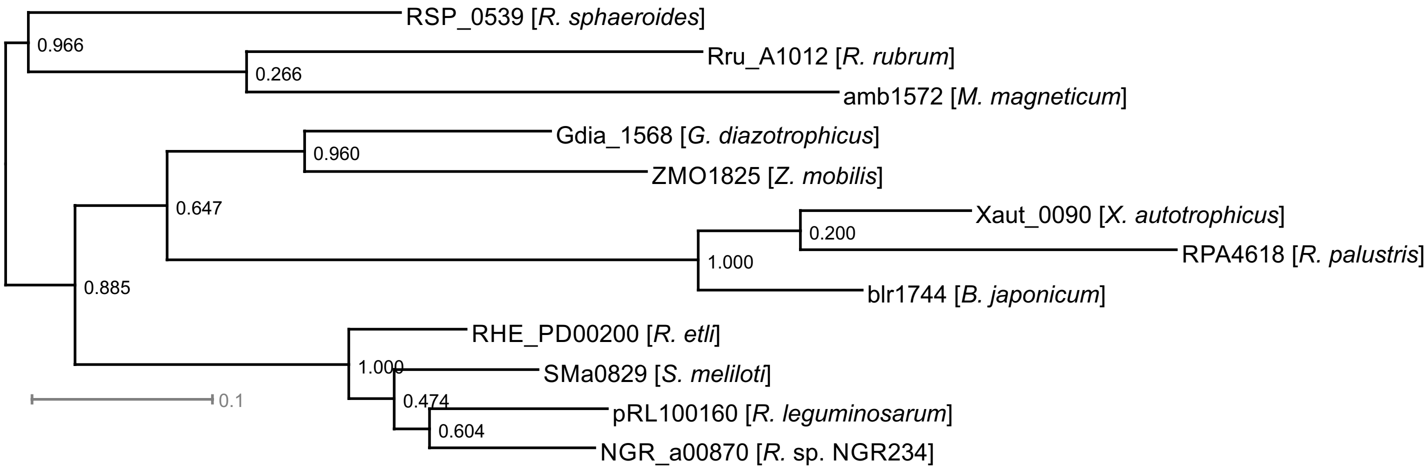

(G) **NifN**

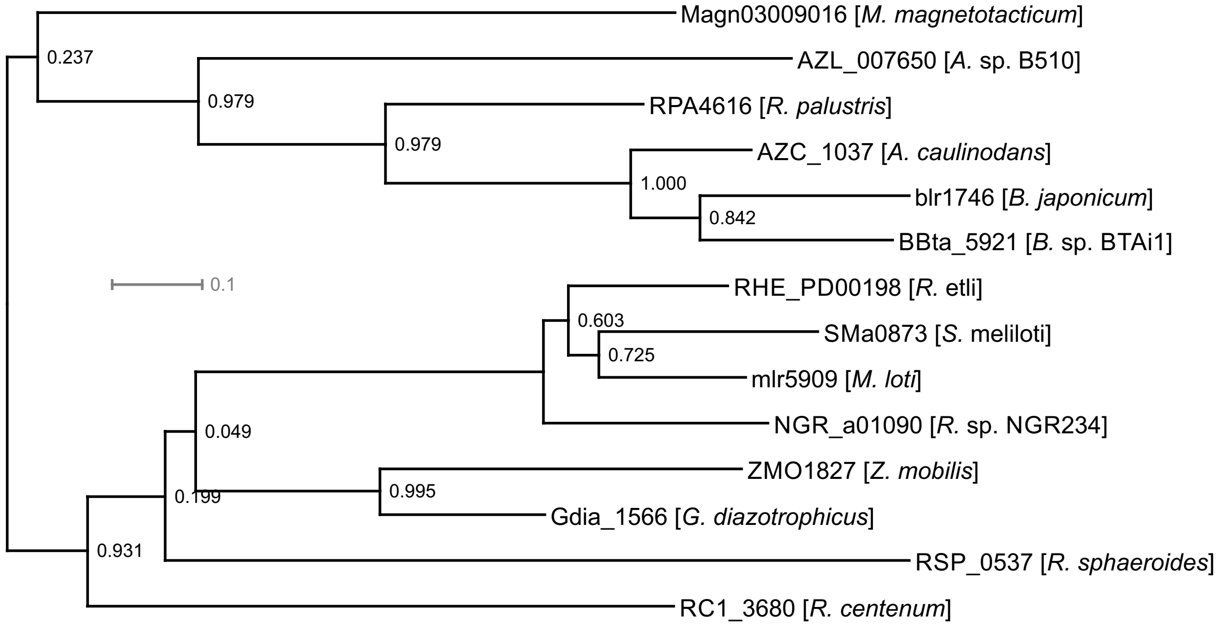

(H) **NifS**

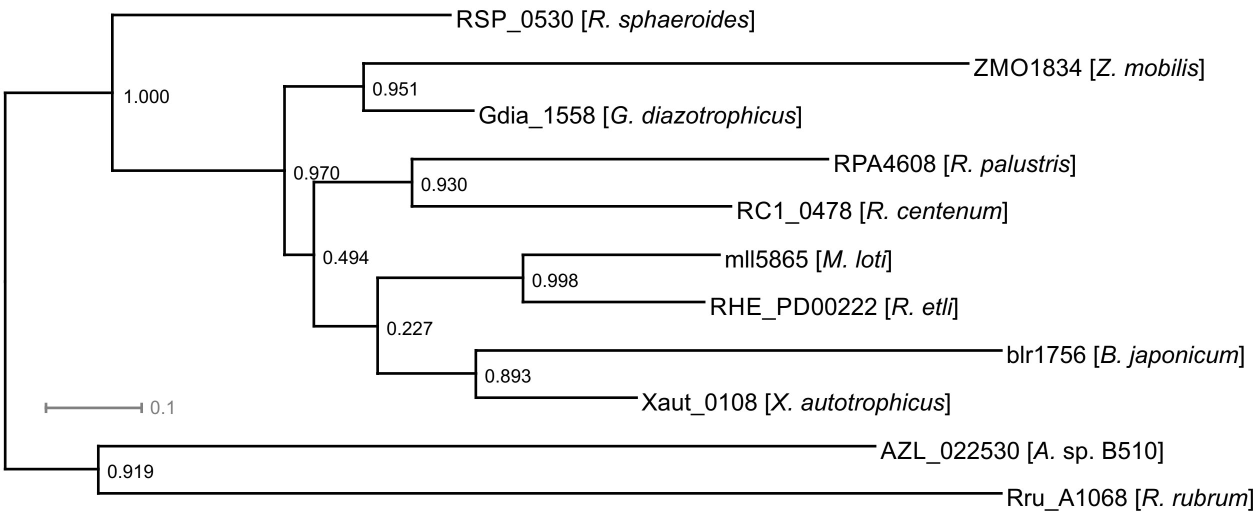

(I) **NifV**

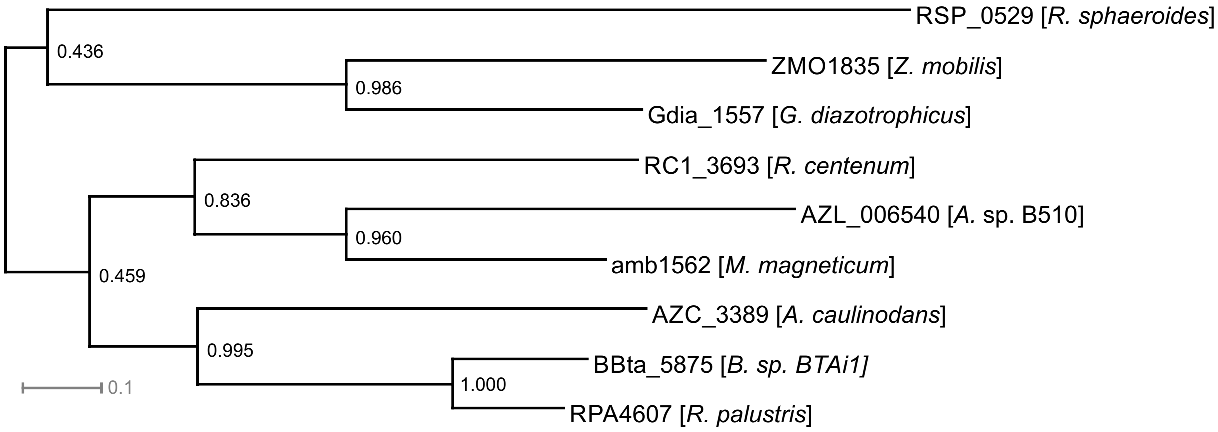

(J) **ModD**

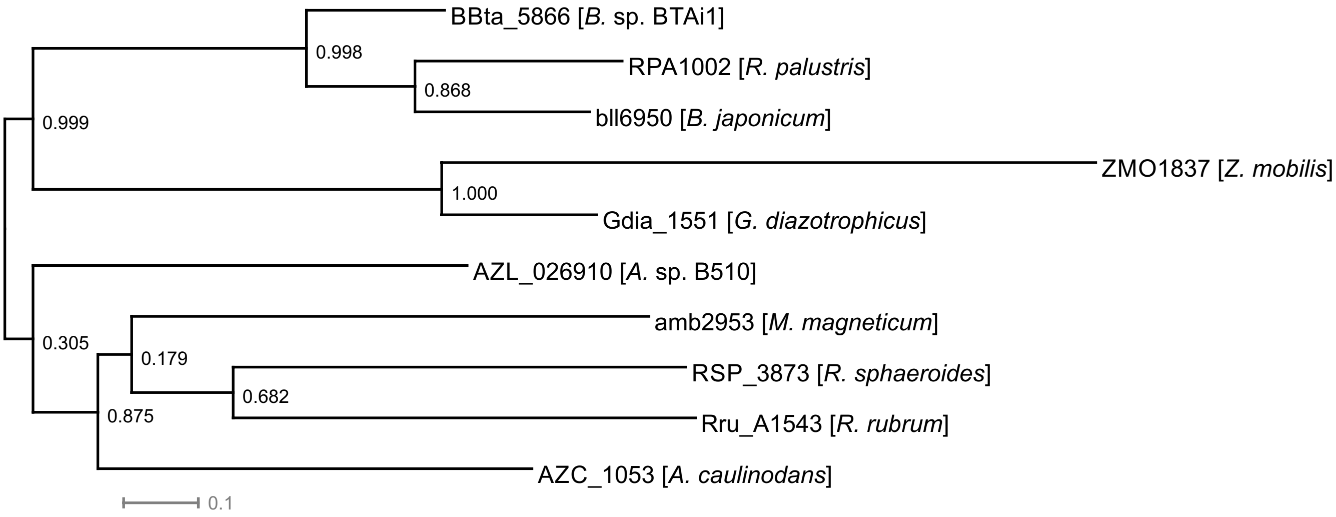

(K) **CydA**

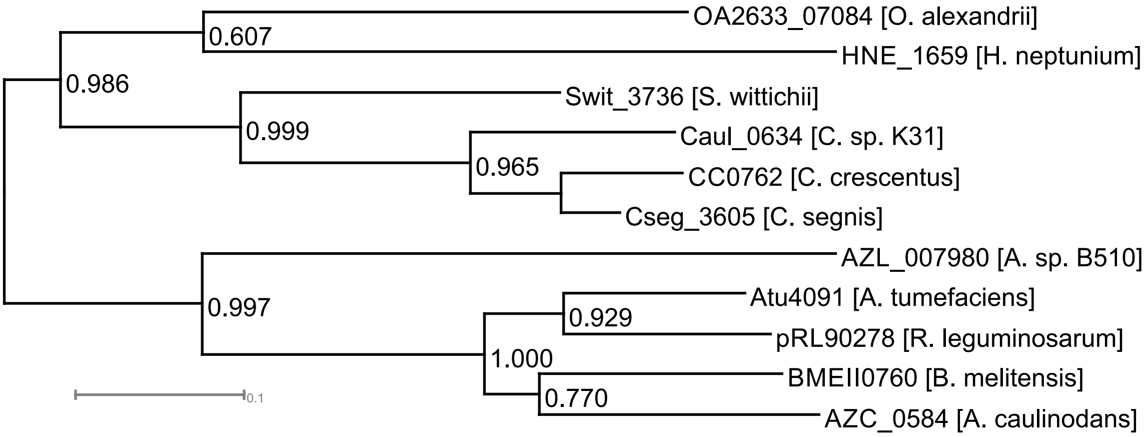

(L) **CydB**

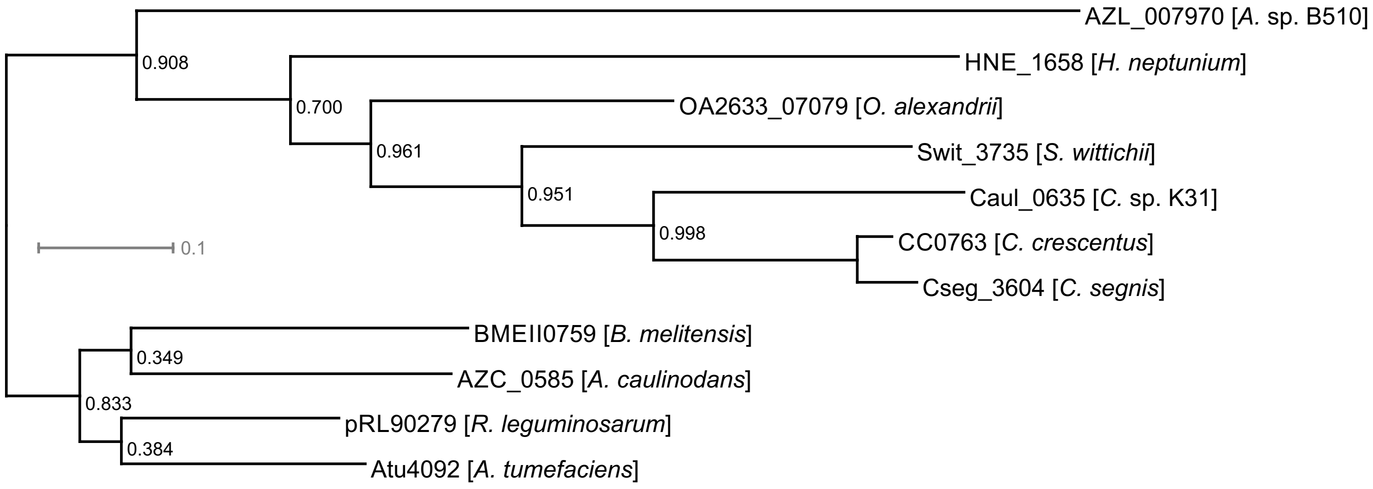

(M) **CydC**

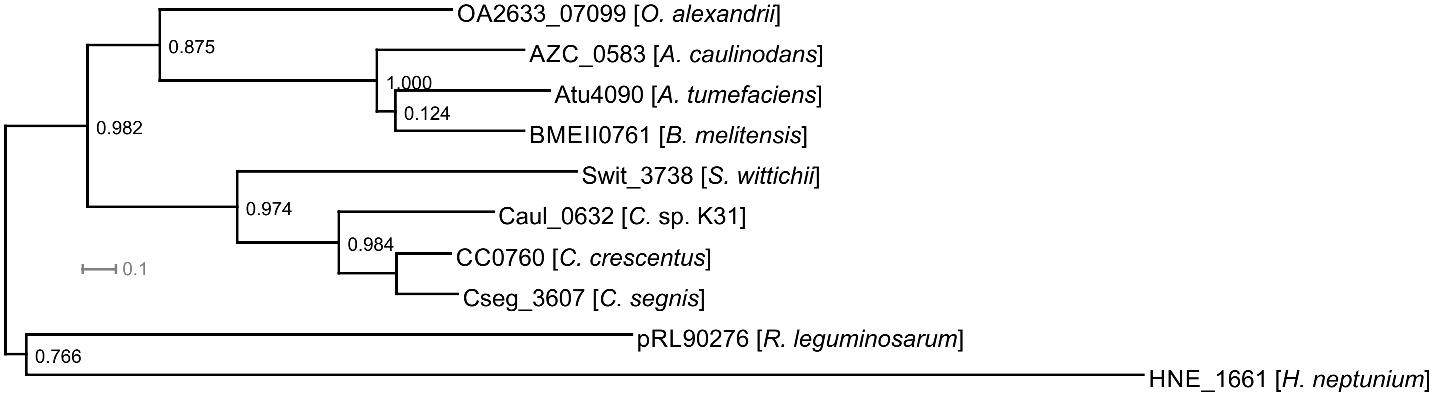

(N) **CydD**

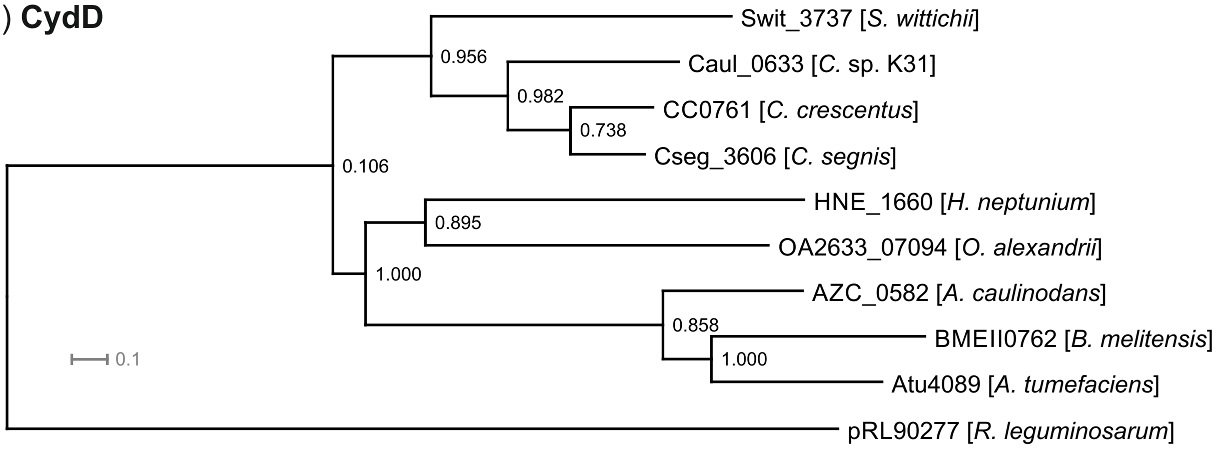

Supplement: Supplementary file 9 [file Image_5.PDF]
